# ROS is a master regulator of *in vitro* matriptase activation

**DOI:** 10.1371/journal.pone.0267492

**Published:** 2023-01-30

**Authors:** Darius O. Gaymon, Robert Barndt, Hillary Stires, Rebecca B. Riggins, Michael. D. Johnson

**Affiliations:** Department of Oncology, Lombardi Comprehensive Cancer Center, Georgetown University, Washington, DC, United States of America; Universita degli Studi della Campania Luigi Vanvitelli, ITALY

## Abstract

Matriptase is a type II transmembrane serine protease that is widely expressed in normal epithelial cells and epithelial cancers. Studies have shown that regulation of matriptase expression and activation becomes deranged in several cancers and is associated with poor disease-free survival. Although the central mechanism of its activation has remained unknown, our lab has previously demonstrated that inflammatory conditions such as intracellular pH decrease strongly induces matriptase activation. In this investigation, we first demonstrate clear matriptase activation following Fulvestrant (ICI) and Tykerb (Lapatinib) treatment in HER2-amplified, estrogen receptor (ER)-positive BT474, MDA-MB-361 and ZR-75-30 or single ER-positive MCF7 cells, respectively. This activation modestly involved Phosphoinositide 3-kinase (PI3K) activation and occurred as quickly as six hours post treatment. We also demonstrate that matriptase activation is not a universal hallmark of stress, with Etoposide treated cells showing a larger degree of matriptase activation than Lapatinib and ICI-treated cells. While etoposide toxicity has been shown to be mediated through reactive oxygen species (ROS) and MAPK/ERK kinase (MEK) activity, MEK activity showed no correlation with matriptase activation. Novelly, we demonstrate that endogenous and exogenous matriptase activation are ROS-mediated *in vitro* and inhibited by N-acetylcysteine (NAC). Lastly, we demonstrate matriptase-directed NAC treatment results in apoptosis of several breast cancer cell lines either alone or in combination with clinically used therapeutics. These data demonstrate the contribution of ROS-mediated survival, its independence of kinase-mediated survival, and the plausibility of using matriptase activation to indicate the potential success of antioxidant therapy.

## Introduction

Matriptase is a type II transmembrane serine protease that is widely expressed by the epithelial components of essentially all tissues. Matriptase was first identified in the conditioned media of T47D human breast cancer cells [1] and subsequently purified from human milk as a 95kDa complex with the Kunitz-type protease inhibitor hepatocyte growth factor activator inhibitor-1 (HAI1) [2]. Matriptase is expressed as an inactive zymogen, with normal synthesis and trafficking to the cell surface dependent on the chaperone-like functions of the primary cognate inhibitor HAI-1, or the closely related Kunitz inhibitor HAI-2 [3]. Forced expression of matriptase in cells lacking HAI expression results in low-level expression of the protein that does not efficiently traffic from the Golgi Apparatus [4, 5]. In normal epithelial tissues, matriptase activation is very tightly regulated, resulting in very low levels of active enzyme generated under strict spatial and temporal control [6]. Unlike the majority of serine proteases, matriptase activation does not require the action of another active protease to mediate proteolytic cleavage at the canonical activation site and instead proceeds through an incompletely characterized auto-activation mechanism [4, 7]. The induction of auto-activation is tightly regulated, but can be initiated or enhanced by a variety of cell-type specific and more universal stimuli. These include the actions of sphingosine 1-phosphate (S1P), which can induce activation in normal mammary epithelial cells [8], androgen which can induce matriptase activation in androgen-dependent prostate cancer cells [9, 10], or apparently universal stimuli such as exposure to a mildly acidic extracellular environment [11]. In epithelial cells and carcinomas, matriptase activation is followed almost immediately by the inhibition of the activity of the protease by binding to HAI-1, which is generally expressed at large molar excess relative to matriptase [12]. Activated matriptase has a role in the maintenance of the epithelial integrity [13]. Knockout of the mouse matriptase homologue, epithin, results in perinatal death that results from aberrations in skin barrier function leading to dehydration [14], and patients lacking functional matriptase expression exhibit problems including ichthyosis [3] and developmental abnormalities [14, 15].

Matriptase can act as a carcinogen and/or tumor promoter in some contexts. For example, modest overexpression of matriptase in the epidermis of mice causes spontaneous transformation and increased DMBA-induced carcinogenesis [16], and many of the known down-stream substrates activated by matriptase are thought to play a role in tumor progression such as pro-HGF and PAR2 [17, 18]. As noted above, matriptase activation is very tightly regulated with very low levels of active enzyme detectable in normal tissues [19]. In epithelial tumors, however, while still inducible, matriptase activation is somewhat constitutive and conditions that favor enhanced matriptase activation and activity have been associated with poor outcome in almost all carcinoma’s examined [20]. We are interested in the mechanisms underlying the altered regulation of matriptase activation in cancer and in particular factors that relate to the tumor microenvironment, such as low pH and inflammatory redox conditions have been shown to activate matriptase [6, 11].

About 1 in 8 women in the US are expected to develop invasive breast cancer in her lifetime [21]. Breast cancers are categorized by the presence or absence of hormone or cell surface receptors such as the estrogen receptor (ER) and Human Epidermal Growth Factor-2 (HER2). These women are routinely treated with targeted therapy that antagonize hormone estrogen signaling such as Fulvestrant or 4-Hydroxy Tamoxifen [21], or Trastuzumab/Pertuzumab and Lapatinib, which inhibit HER2 activity. Additionally, breast tumors without ER expression or HER2 overexpression or amplification are categorized as Triple Negative Breast Cancer (TNBC). TNBC tumors often express the androgen receptor (AR) and currently only treated with chemotherapy [22]. While treatment options have increased in specificity and effectiveness over the years, resistance is common and nearly 45,000 women are expected to succumb to the disease in 2021. Many of the resistance mechanisms are unknown which demonstrates the need for a deeper understanding of the survival capability of cancer cells. While it is clear that major signaling pathways such as the ER and HER2 pathways are major regulators in various cancers, it has also been shown that other signaling molecules, such as reactive oxygen species (ROS) are important in regulating cell fates. Normal cell growth requires anabolic processes that are driven by ATP and mitochondrial metabolism, the source of ATP, also causes the production of superoxide and hydrogen peroxide, a major type of ROS, as a byproduct [23, 24]. In normal cell biology, low levels of ROS are required to maintain stem cell quiescence and self-renewal while increases cause proliferation, differentiation, as well as apoptosis [25]. Several intracellular antioxidants such catalase, NADPH, and glutathione help regulate ROS production or availability. Tumor cells, due to an increased growth and proliferation rates, have an increase in anabolic processes as well as ATP and ROS production [26]. Basal ROS levels are increased in several cancers [23] and the range of ROS increase has been shown to have a variety of cellular effects. Additionally, ROS has been shown to intimately involved in the dynamics of estrogen-induced transcription. A recent publication has shown ROS production in two phases following estrogen challenge, demonstrating its role as a secondary messenger in estrogen signaling [27]. Another publication demonstrates estrogen challenge induces transcription mediated by the demethylation of lysine 9 in histone (H3K9) and followed by the generation of ROS that oxidizes nearby Guanines into 8-oxo-Guanines. The hormone induced phosphorylation of serine 10 in histone 3 (H3S10) prevents remethylation of H3K9 and allows for proper DNA repair of the oxidized Guanines. Inhibition of H3S10 in ER-positive MCF7 cells resulted in an overproduction of ROS and a switch to cell death [28]. Therefore, it is critical to fully understand the contribution of ROS to the network of pathways resulting in determining cell fates of cancer cells.

In this study, we show that endogenous and exogenous matriptase activation are ROS-mediated *in vitro* and we further demonstrate matriptase activation may serve as an indicator of NAC efficacy in inducing cell death in several breast cancer subtypes.

## Methods

### Cell culture

All breast cancer cell lines were obtained from the Lombardi Comprehensive Cancer Center (LCCC) Tissue Culture & Bio-specimens Shared Resource (TCBSR). MCF7 and MDA-MB-468 cells were grown in IMEM (Gibco, Gaithersburg, MD) supplemented with 10% FBS (TCBSR). SKBR3 and MDA-MB-361 cells were grown in DMEM (Lonza, Walkersville, MD) supplemented with 10% FBS. HCC1569 andAU565, were grown in RPMI 1640 (Lonza) supplemented with 10% FBS. Cells were cultured in a humidified incubator at 37C in air with 5% CO2. The cells were tested for mycoplasma contamination (last date: April, 2017) and their identity was confirmed by short tandem repeat profiling conducted by the LCCC TCBSR. For biochemical experiments cells were typically plated at approximately 80% confluence and then subsequently treated with the indicated concentrations of reagents or vehicle controls for the indicated periods. For some experiments, cells were serum starved for the periods indicated, by washing the cell monolayers with 1X sterile PBS and then replacing the medium with serum free medium, alone or in combination with the indicated drugs. Conditioned medium and lysates were prepared as described below.

### Immunoblot assays and antibodies

Lysates for immunoblot assays to assess the level and activation state of matriptase were prepared lysing the cells in 1% Triton X-100 in Phosphate Buffered Saline (PBS) with 1mM DTNB (5,5-dithio-bis-(2-nitrobenzoic acid)). The addition of DTNB in the lysis buffer is to prevent the cleavage of disulfide linkages [29], which is important to preserve activated matriptase complexes with HAIs, particularly for the samples containing reducing species, such as reduced glutathione in the cell lysates. The lysates were centrifuged at 12,000 rpm for 2 minutes and the supernatants collected. Protein concentrations in the lysates were determined by Protein Assay solution (BioRad, Hercules, CA) with reference to a bovine serum albumin standard curve. Equal amounts of total protein were mixed with Laemmli sample buffer lacking any reducing agent and incubated at room temperature for 5 minutes (non-reduced, non-boiled). Protein samples were resolved by 7.5% SDS-polyacrylamide gel electrophoresis (SDS-PAGE) under non-reducing and non-boiled conditions to preserve activated matriptase complexes with HAIs, transferred to either nitrocellulose or PVDF membrane, and subsequently probed with mAbs, as indicated. Immunoreactive regions were visualized using horseradish peroxidase-labeled secondary antibodies and Western Lightning ECL pro reagent (PerkinElmer, Waltham, MA), and imaged using the Amersham Imager 600 digital system (VWR, Radnor, PA). The primary antibodies used were the total matriptase mAb M24, the activated matriptase mAb M69, and the HAI-1 mAb M19. The generation and characterization of which can be found in our previous studies [16, 30]. The total matriptase antibody binds to inactive zymogen and activated forms of matriptase including activated complex. The activated matriptase antibody only binds to the activated form of matriptase, including activated complex.

Media conditioned by culture with the cells in some experiments were also collected and analyzed for the level and activation state of the matriptase species shed from the cells. Equal volumes of medium directly from the cells, or concentrated as indicated, were mixed with 5X loading buffer and analyzed as described above.

Immunoblot assays for EGFR family members and downstream signaling molecules were conducted using samples prepared in parallel with those used for matriptase assays due to incompatibilities in the lysis buffers. Lysates for immunoblots of signaling molecules were prepared using lysis buffer (10 mM Tris-base, pH 7.4, 1% Triton X-100, 50 mM NaCl, 30 mM sodium pyrophosphate, 50 mM sodium fluoride, 1 mM sodium orthovanadate, 5 mM β-glycerophosphate, 1 mM phenylmethylsulfonyl fluoride, and 2 μg/ml each of pepstatin, leupeptin, and aprotinin) as previously described [30]. Protein concentrations were determined by BioRad assay as above, and samples containing equal amounts of protein were mixed with sample buffer containing Beta-mercaptoethanol and boiled for 5 minutes prior to separation by SDS-PAGE and transferred to membranes as above. Immunoblot assays for the indicated proteins were conducted using the following antibodies at 1:1000 dilution; Total EGFR Cat. No. 4267S (Cell Signaling, Danvers, MA), p-Y1068 EGFR Cat. No. 2234 (Cell Signaling), Total HER2 Cat. No. 4290 (Cell Signaling), p-Y877 HER2 Cat. No. 2241 (Cell Signaling), Total MAPK Cat. No. 4695 (Cell Signaling), p-P44/42 MAPK Cat. No. 4370 (Cell Signaling), Pan AKT Cat. No. 4685 (Cell Signaling), p-S473 AKT Cat. No. 4060 (Cell Signaling), and GAPDH Cat. No. 5174 (Cell Signaling). Total matriptase (M24-TM) and HAI-1 (M19) were incubated at 1:1000 while activated matriptase (AM) was incubated at 1:500 and have been characterized in our previous publication [19].

### Annexin V assay

100,000 cells/well were seeded in 6-well plates and allowed to attach overnight. The next day, cells were treated in serum-replete conditions with the appropriate pharmacological agents and allowed to incubate for 4 days. NAC treatments occurred on days 1 and 3. Dead cells were collected with conditioned media and live cells were trypsinized. Both were centrifuged together for 5min at room temperature, re-suspended in 1ml serum-replete media, and given to the flow cytometry core at Lombardi Cancer Center for assay. The cells were stained with Annexin-FITC and propidium iodide and gated based on FSC/SSC size scatter to exclude debris and aggregates.

### Statistics

To test for outlier comparisons between treatment groups, two-way and three-way ANOVA will be used, where appropriate. To test for statistical significance between treatment groups, student’s t-tests with Welch’s correction were run by GraphPad Prism software. P values of .05 or less were considered statistically significant;* p<0.05, ** p<0.01, ***p<.0.001.

## Results

### Investigating activated matriptase levels demonstrated that ICI and Lapatinib treatment result in robust activation in double positive breast cancer cells

**Fig 1:**
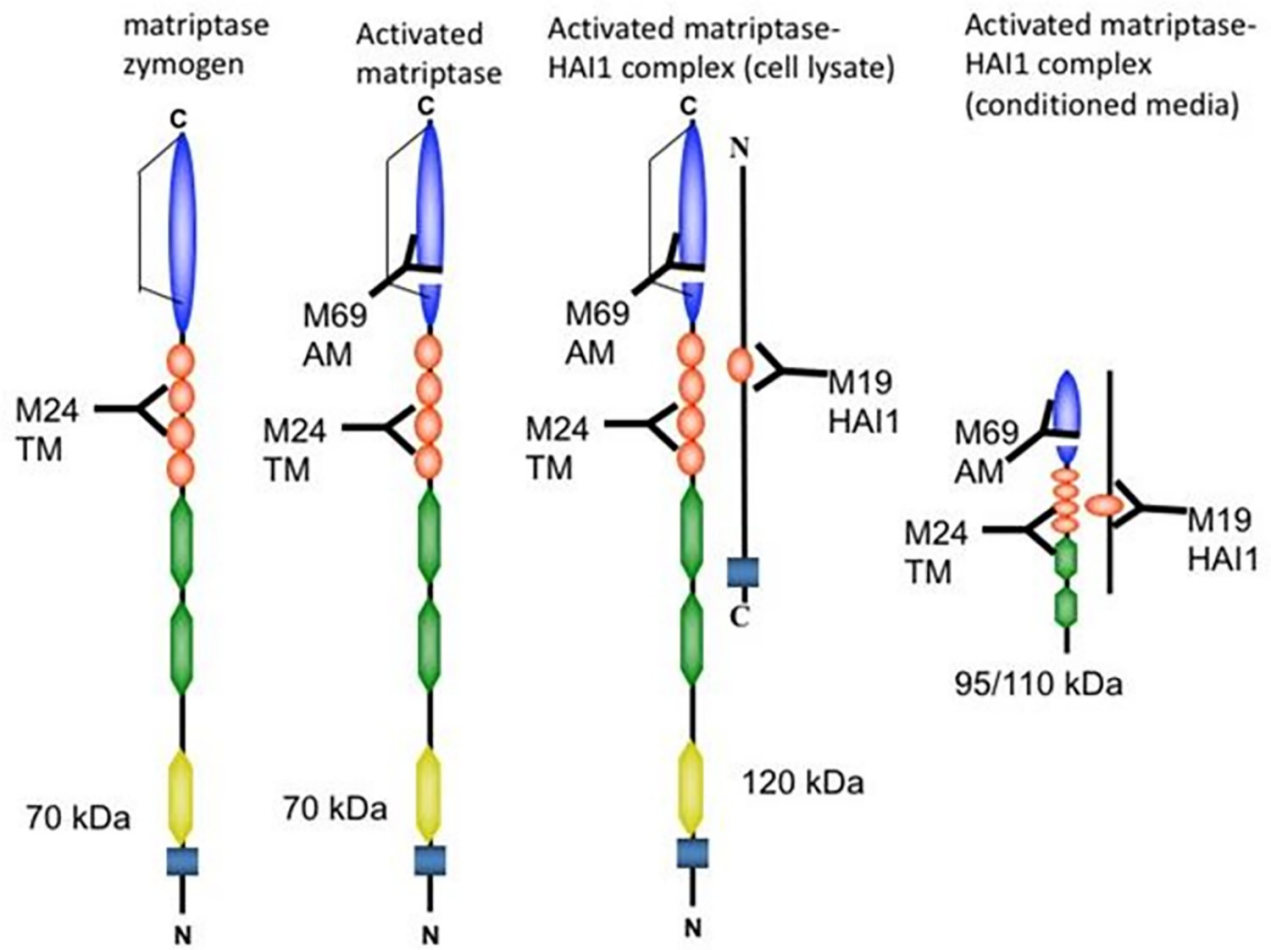
Matriptase expression and activation states.

**Fig 2:**
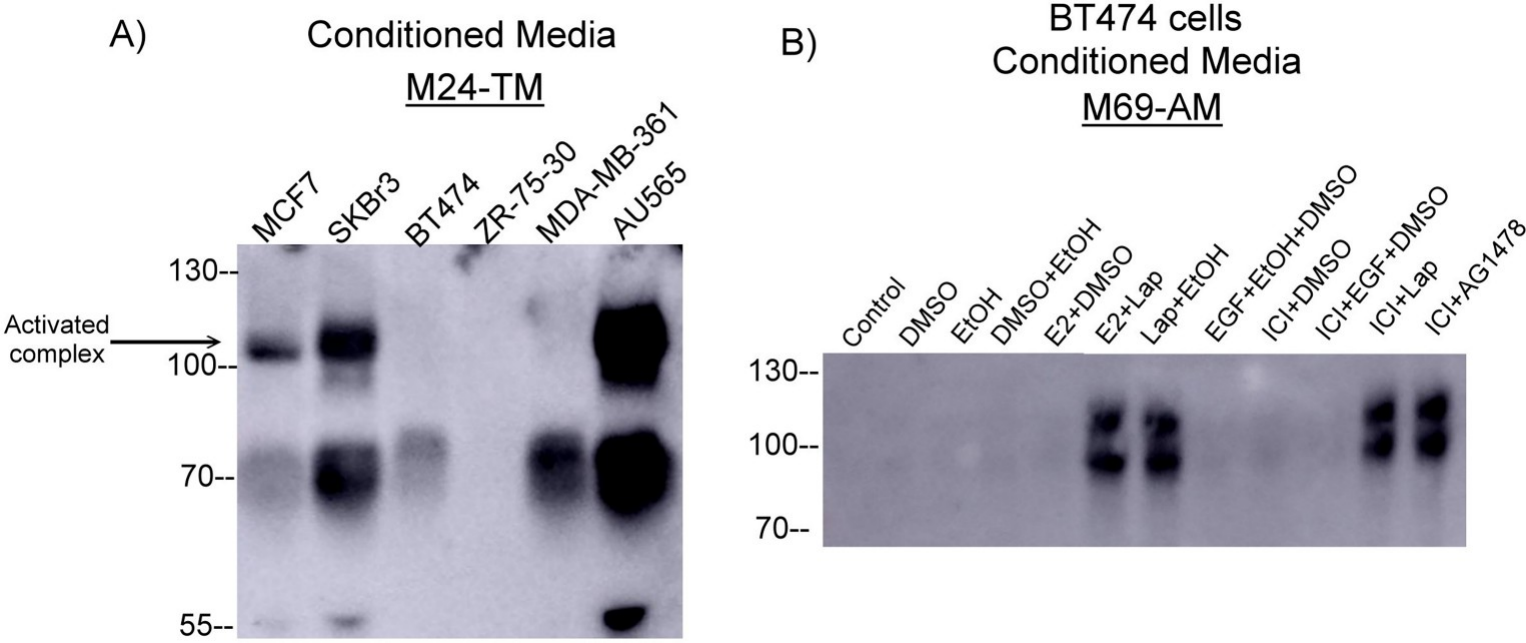
Screening breast cancer cells revealed robust matriptase activation only when ER-positive cells are treated with ICI and Lapatinib. A) Breast cancer cells were seeded in 35mm dishes and when ~80% confluent, incubated for 24 hours in 1ml of basal media + 10% FBS. Conditioned media was collected and the level of activated matriptase was determined by immunoblotting with the total matriptase antibody. B) BT474 cells were seeded in 35mm dishes and when ~80% confluent, washed twice with sterile PBS, and pre-treated with 1uM ICI in serum-free media for 48 hours. Cells were then treated with either 20ng/ml EGF, 10nm estradiol (E2), 1uM ICI, or 5.4uM Lapatinib (Lap) with appropriate solvent controls for 48 hours. Conditioned media was collected and the level of activated matriptase was determined by immunoblotting with the activated matriptase antibody. Panels were the highest quality representative of 4 biological replicates.

Matriptase is first expressed as a zymogen and activated as a result of an autocleavage in its serine protease domain. The final step in matriptase activation is cleavage and liberation of the activated complex, which can be detected via immunoblot (Fig 1). We first set out to investigate baseline levels of matriptase activation using a panel of well-known HER2-amplified and ER-positive breast cancer cells by probing for activated matriptase-HAI1 complex under basal conditions in serum-containing media. The HER2-amplified cells are SkBr3 and AU565. MCF7 are ER+ while BT474, MDA-MB-361, and ZR-75-30 are both ER+ and HER2-amplified (double positive). In ER+ MCF7 cells, which we have previously demonstrated to have ‘leaky’ matriptase activation, we demonstrate the presence of the ~110 activated matriptase complex in 24-hour conditioned media (Fig 2A). Little to no shed 95- and 110-kd matriptase-HAI1 complexes were detected in 24-hour standard conditioned media from double positive BT474, MDA-MB-361, and ZR-75-30 cells. In comparison, HER2-amplified AU565 and SKBr3 cells showed clear baseline activation of matriptase as observed in shed 95- and 110-kd matriptase-HAI1 complexes (Fig 2A). We next aimed to determine if stimulation of ER or HER2 could increase levels of matriptase activation in double positive cells. Double positive BT474 cells pretreated with 1uM ICI in serum-free media for 24 hours to attenuate hormone and growth factor signaling before treating with 20ng/ml EGF, 1nM estradiol, 5.4uM Lapatinib (HER2/EGFR tyrosine kinase inhibitor), or continuing ICI for an additional 24 hours. Fig 2B shows no increase in matriptase-HAI1 complex levels as a result of 24-hour stimulation with vehicle controls or estradiol alone. However, we observed a large induction of matriptase activation demonstrated by the formation and detection of activated matriptase complex resulting from 24-hour Lapatinib treatment both in the presence and absence of estradiol (Lanes 6 and 7). Neither EGF nor ICI treatment alone or in combination resulted in an increase in complex levels and similar to lanes 6 and 7, Lapatinib or the EGFR Tyrphostin, AG825, in combination with ICI resulted in the formation and shedding of activated matriptase. This and future immunoblots are assessments of conditioned media and are normalized by loading volume (15ul) in each well. Loading controls such as GAPDH cannot be used for analysis of activated and shed matriptase in conditioned media.

### Investigating ICI and Lapatinib induced matriptase determined that only modest activation resulted from PI3K inhibition with no activation from MAPK inhibition and occurs as early as six hours post treatment

**Fig 3:**
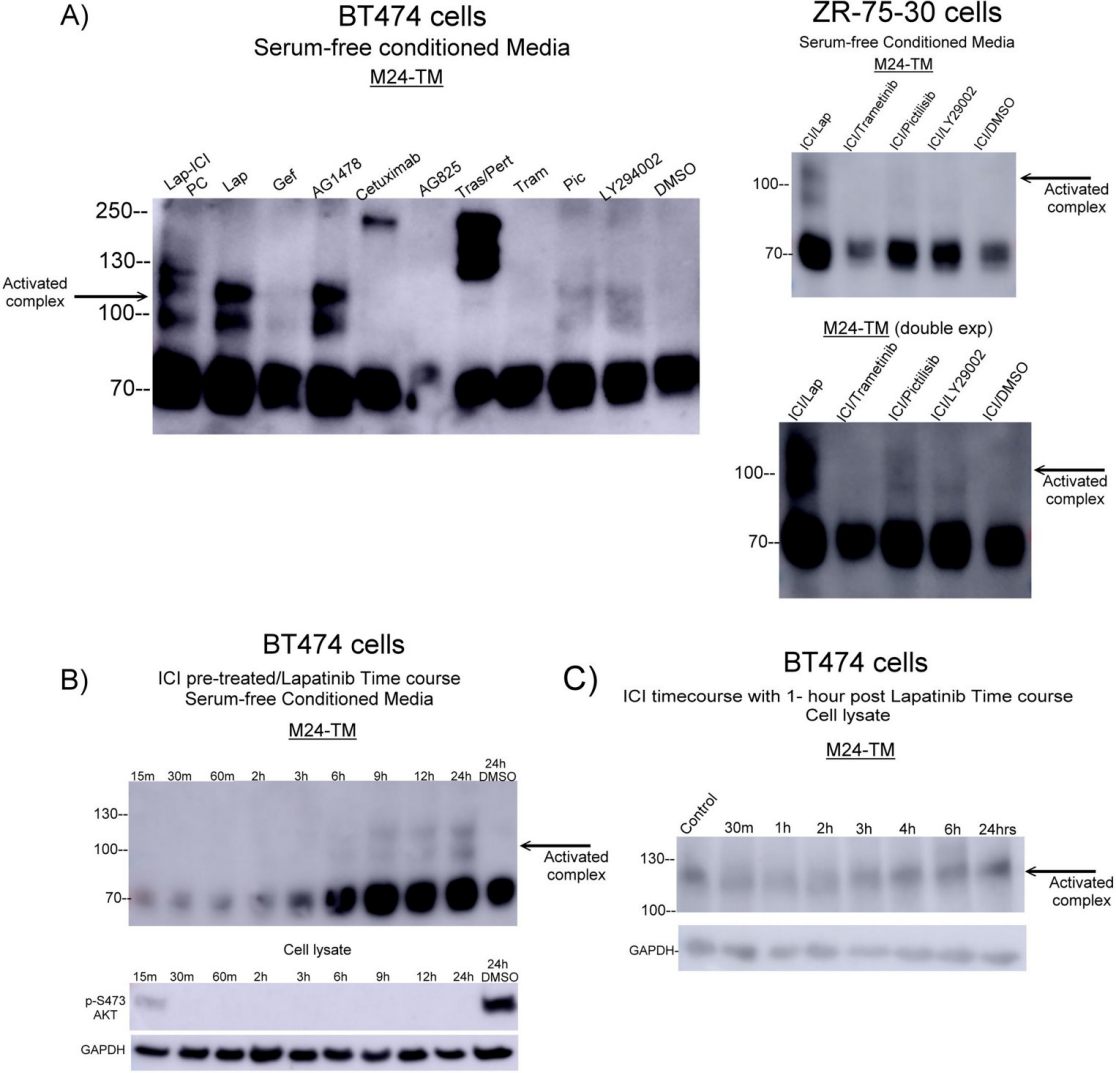
Investigating ICI and Lapatinib induced matriptase determined that only modest activation resulted from PI3K inhibition with no activation from MAPK inhibition and occurs as early as six hours post treatment. A) BT474 and ZR-75-30 cells were seeded into 35mm dishes and when ~90% confluent, washed twice with sterile 1X PBS and incubated for 24 hours in serum-replete media with 1uM ICI. Cells were then treated with 5.4uM Lapatinib, 10uM Trametinib, 10uM Pictilisib, or 0.01% DMSO. Conditioned media was collected with the appropriate lysis buffer (see methods). B) BT474 cells were seeded into 35mm dishes and when ~90% confluent, washed twice with sterile 1X PBS and incubated for 24 hours in serum-replete media with 1uM ICI. Cells were then spiked with 5.4uM Lapatinib for increasing amounts of time. Cell lysates and conditioned media were collected with the appropriate lysis buffer. The levels of phospho-AKT, total and activated matriptase were determined by immunoblotting with p-S473 AKT and total matriptase (M24) monoclonal antibodies. C) BT474 cells were seeded into 35mm dishes and when ~90% confluent, washed twice with sterile 1X PBS and incubated for increasing amounts of time in serum-free media with 1uM ICI. After each time point, cells were spiked with 5.4uM Lapatinib for 1 hour. Cell lysates were collected with the appropriate lysis buffer and the levels of total and activated matriptase were determined by immunoblotting with the total matriptase (M24) monoclonal antibody. GAPDH was used as a loading control. Panels were the highest quality representative of 4 biological replicates.

We next asked whether Lapatinib-induced matriptase activation involved downstream signaling through PI3K or MAPK [31]. Following the same experimental design as described above, in BT474 and ZR-75-30 cells we compared the levels of matriptase activation induced by ICI pretreatment and subsequent 1uM Trametinib (MEK inhibition) and 1uM Pictilisib (PI3K inhibition). In cell lines, PI3K inhibition, but not MAPK inhibition, modestly induces matriptase activation (Fig 3A). When compared to Lapatinib, the level of activated matriptase-HAI1 complex was lower in Pictilisib-induced BT474 and ZR-75-30 cells, suggesting that other downstream signaling pathways contribute to lapatinib-induced matriptase activation. We next determined the time course of ICI and Lapatinib-induced matriptase activation. We first pretreated BT474 cells with 1uM ICI for 24 hours and then, incubated with 5.4uM Lapatinib for increasing amounts of time. We were able to appreciably detect matriptase-HAI1 complex in conditioned media by 6 hours, which continued to accumulate through the last time point of 24 hours (Fig 3B). Next, we attempted an ICI time course with a shortened 1 hour-post Lapatinib treatment and in cell lysates and observed a consistent increase in 120kd matriptase-HAI1 complex by 6 hours ICI treatment (Fig 3C).

### Comparing anticancer agent treatments demonstrated that matriptase activation was not a hallmark of each treatment but was robustly induced by etoposide

**Fig 4:**
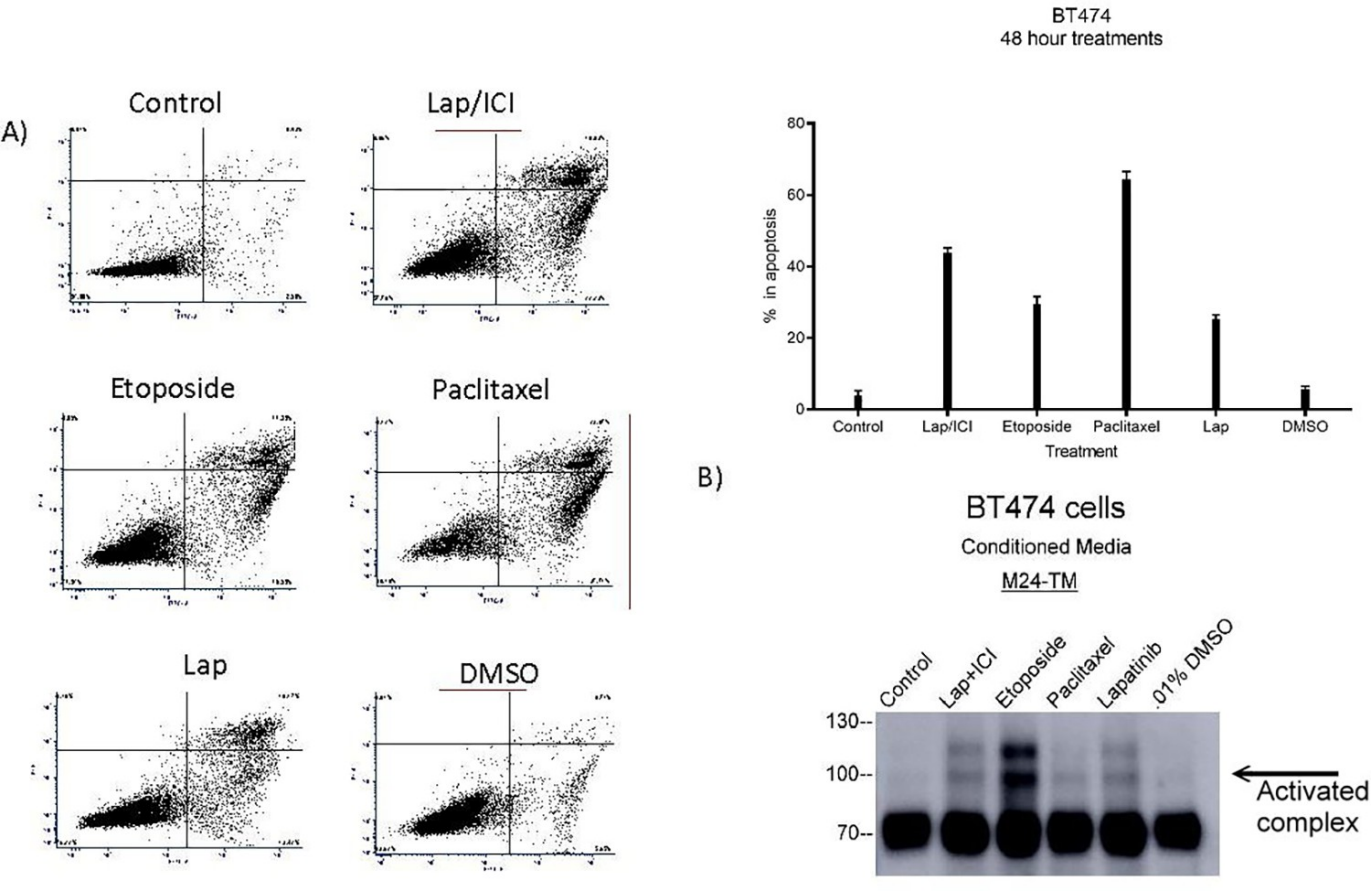
Comparing methods of cellular stress showed that matriptase activation was not a hallmark of cellular stress but was robustly induced by etoposide. 100,000 BT474 cells/well were seeded into wells of a 6-well plate and allowed to attach overnight. Cells were then treated with 5.4uM Lapatinib and 1uM ICI in combination, 10uM Etoposide, 1uM Paclitaxel, 5.4uM Lapatinib alone, or 0.01% DMSO in either serum-replete or serum-free parallel experiments. Panels are representative of three biological replicates. Cells treated in serum-replete conditions were collected and A) assayed for apoptosis by Annexin V/Propidium Iodide staining and the conditioned media from cells treated in serum-free conditions was collected in order to B) determine levels of total and activated matriptase by immunoblotting with the total matriptase (M24) monoclonal antibody. Panels were the highest quality representative of 4 biological replicates.

As ICI and Lapatinib are both clinically used therapies [32] known to induce cellular stress and/or apoptosis in various cancer cell models [29, 30], we next investigated if matriptase activation could be a hallmark of cellular stress or apoptosis. We compared the levels of matriptase activation in BT474 cells treated with ICI and Lapatinib to cells treated with 1uM Etoposide, 1uM Paclitaxel, and 5.4uM Lapatinib alone. Dual experiments were completed in parallel in order to assess levels of matriptase activation in serum-free media and levels of apoptosis in serum-replete media. Etoposide and Paclitaxel were chosen because of their ability to induce different pathways of cellular stress and apoptosis via DNA damage [33] or cell cycle arrest [34], respectively. We observed by Annexin V/Propidium Iodide staining that a similar level of apoptosis was induced by all drug treated conditions, including 48-hour Lapatinib treatment (Fig 4A). We observed no matriptase activation in standard growth and vehicle control conditions while Lapatinib as well as Lapatinib and ICI treated cells induced matriptase activation as previously demonstrated, detected by the total matriptase, M24 antibody. Etoposide treatment resulted in the largest induction of matriptase activation and Paclitaxel treatment induced the lowest level activation matriptase, similar to that of the vehicle control (Fig 4B). The lack of correlation between apoptosis and matriptase activation demonstrated that matriptase activation was not a universal hallmark of apoptosis.

### Investigating endogenous, cell stress, and acid-induced matriptase activation demonstrated those mechanisms were mediated ROS and could be inhibited by N-acetyl cysteine (NAC)

**Fig 5:**
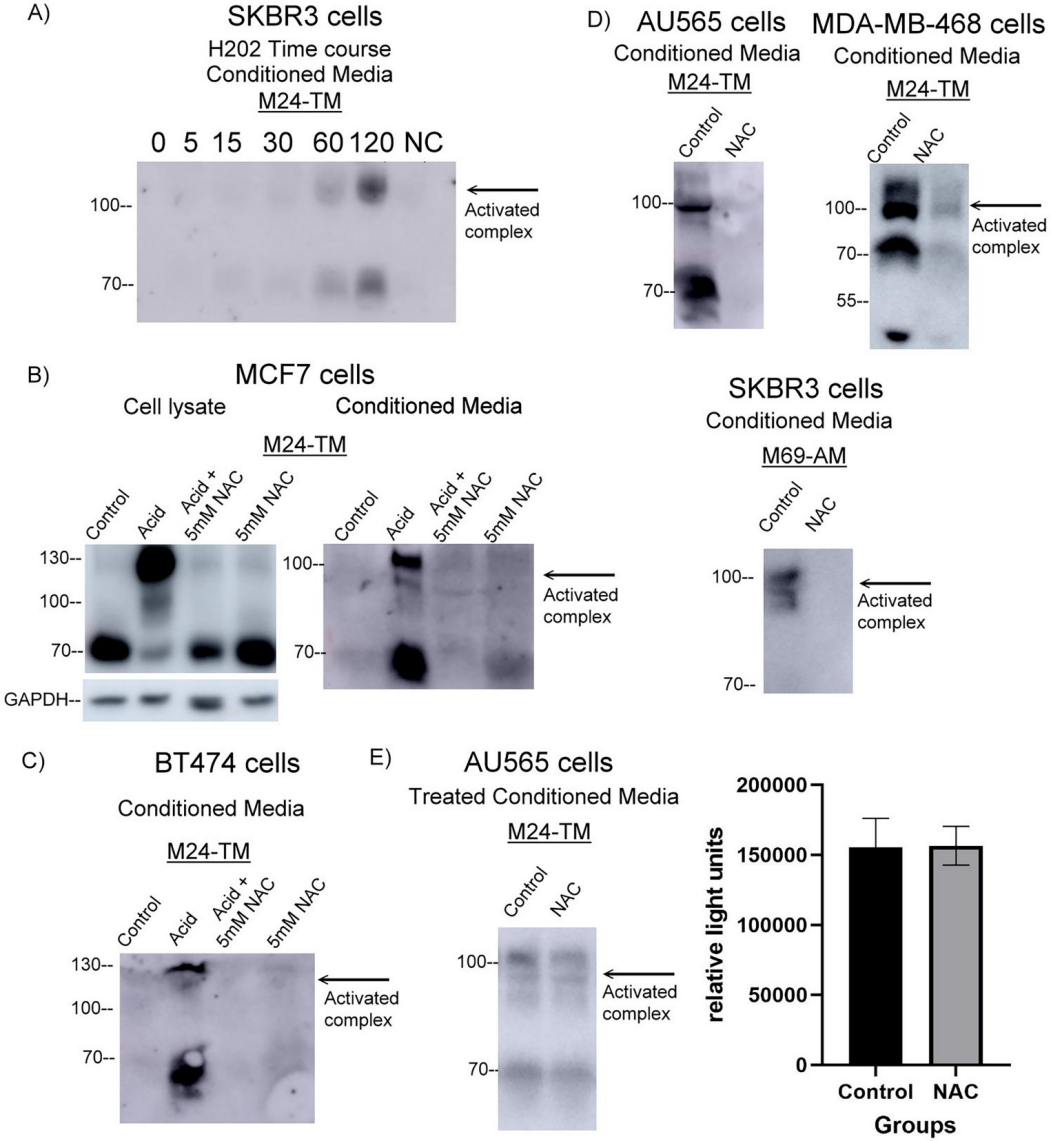
Investigating endogenous, cell stress, and acid-induced matriptase activation demonstrated those mechanisms were mediated ROS and could be inhibited by N-acetyl cysteine (NAC). A) SKBR3 cells were seeded in 35mm dishes and when ~80% confluent, washed twice with 1X sterile PBS and incubated for 3 hours with 5.4uM Lapatinib. Cells were then spiked with 10nm hydrogen peroxide for increasing amounts of time. Conditioned media was collected from the cells and analysed for levels of total and activated matriptase by the total matriptase (M24) antibody. B) MCF7 and C) BT474 cells were seeded in 35mm dishes and when 80% confluent. 5mM NAC was added to serum-replete media for 3 hours to pre-treat the cells. After pre-treatment, acid-induction buffer and 5mM NAC were added to cells either alone and in combination for 20 minutes. Cell lysates and conditioned media were collected in parallel and assayed for GAPDH as well as total and activated matriptase by the GAPDH and total matriptase (M24) antibody. D) MDA-MD-468, AU565, and SKBR3 cells were seeded in 35mm dishes and when ~80% confluent, incubated in 1ml serum-replete media with and without 5mM NAC for 24 hours. Conditioned media was collected from the cells and analysed for levels of total and activated matriptase by the total matriptase (M24) and activated matriptase (M69) antibody. Panels were representative of 3 biological replicates. E) AU565 cells were seeded in 35mm dishes and ~80% confluent, incubated in 1ml serum-replete media. Conditioned media was collected and 5mM NAC was added to experimental samples for 24 hours. The levels of activated matriptase were then assessed using total and activated matriptase antibodies. Panels were the highest quality representative of 4 biological replicates.

It has been recently published that ROS and ERK were two pathways that mediated Etoposide-induced cytotoxicity in human kidney cell [33]. In Fig 3, we demonstrated that ERK (MAPK) inhibition did not induce matriptase activation, which suggested that Etoposide-induced matriptase activation could be ROS dependent. This hypothesis followed previous work from our lab which demonstrated the ability of heavy metals, CoCl_2_ and CdCl_2_, to induce matriptase activation [30]. We first set out to test the ability of hydrogen peroxide to induce matriptase activation in living, SKBR3 cells, by first pre-treating with 5.4uM Lapatinib for 3 hours to suppress native matriptase activation and then co-treating with Lapatinib and hydrogen peroxide for increasing amounts of time. We observed a time-dependent increase of shed, activated matriptase-HAI1 complex in conditioned media following .01% hydrogen peroxide treatment (Fig 5A). In order to further assess the plausibility of ROS as a central mechanism of matriptase activation, we investigated the ability of NAC to inhibit our well-established and previously published acid-induced matriptase activation protocol. This hypothesis seemed likely as we have previously demonstrated NACs ability to inhibit heavy metal induced matriptase activation. In both MCF7 and BT474 cells, where we have demonstrated low levels of endogenous matriptase activation in Fig 2, we first confirmed the ability of a decrease in pH to induce matriptase activation in 20 minutes. Next, we tested NACs ability to directly inhibit matriptase activation by first pretreating MFC7 and BT474 cells with 5mM NAC and then, co-treating with NAC and the previously described acid induction buffer. This method is published to modestly reduce pH, which we have demonstrated to induce matriptase activation [9], however, we have demonstrated that NAC treatment inhibits acid induced matriptase activation in both cell lines while having no effect on matriptase zymogen levels when treated alone (Fig 5B). Next, we wanted to investigate the role of ROS in endogenous matriptase activation using AU565, MDA-MB-468, and SKBR3 cells that were shown to endogenously activate matriptase in standard growth conditions in Fig 1. Cells were treated with 5mM NAC in serum-replete media for 24 hour and conditioned media was assessed for levels of matriptase activation. We have demonstrated that NAC is able to decrease levels of endogenous matriptase-HAI1 complex formation in all three cell lines. Lastly, aimed to determine if NAC treatment could dissociate activated matriptase complex, 5mM NAC was added to AU565 24-hour conditioned media for 24 hours and assessed for levels of matriptase activation. No change in complex levels was observed as a result of incubation of conditioned media with 5mM NAC. Taken together, we have concluded that the acid-induced and endogenous mechanisms of matriptase activation in breast cancer are ROS-dependent *in vitro*.

### Investigating matriptase-directed 5mM NAC treatment resulted in increased apoptosis alone or in combination with ICI and/or Lapatinib in ER-positive breast cancer models

**Fig 6:**
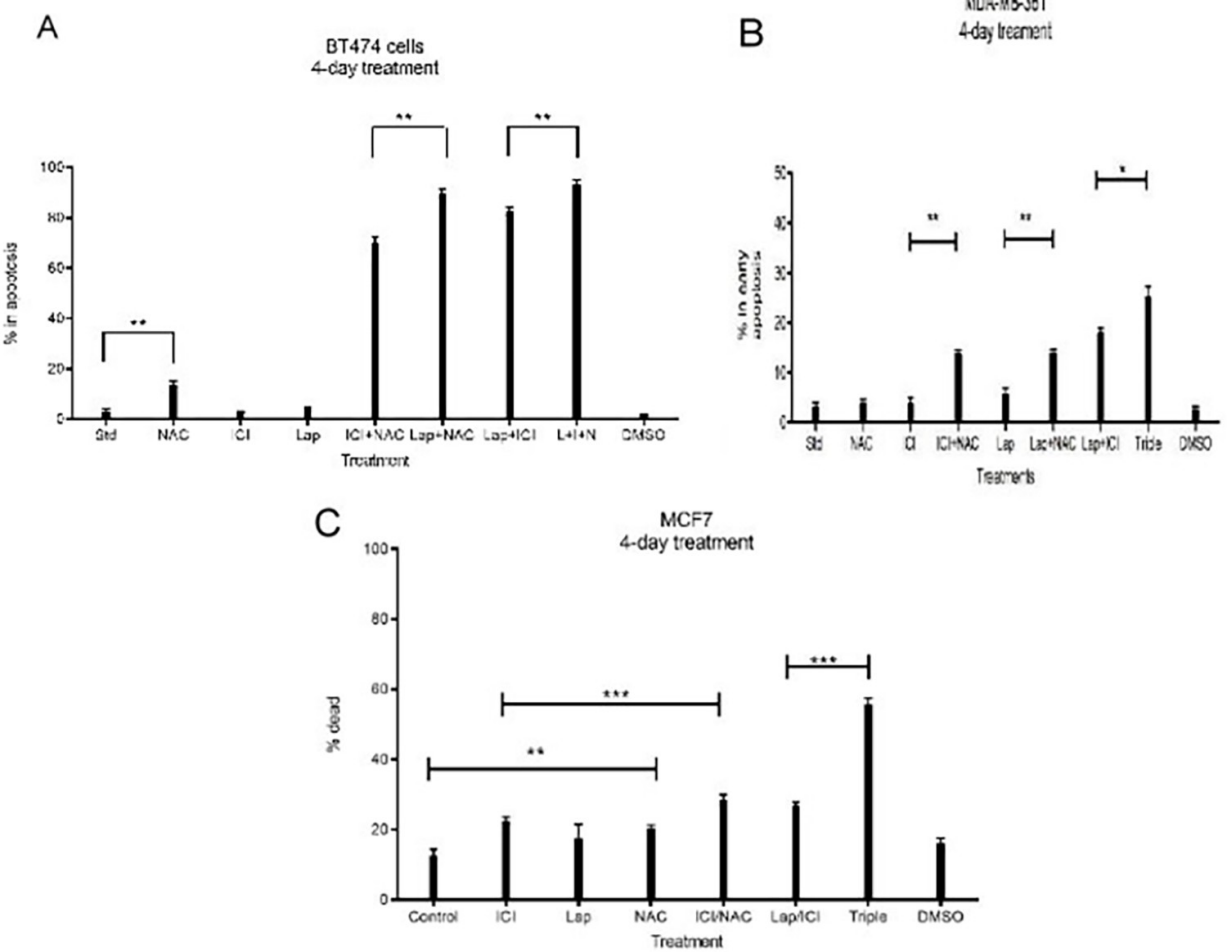
Investigating matriptase-directed 5mM NAC treatment resulted in increased apoptosis alone or in combination with ICI and/or Lapatinib in ER-positive breast cancer models. 100,000 A) BT474, B) MDA-MB-361 and C) MCF7 cells /well were seeded in 6-well plates and allowed to attach overnight. Cells were then treated with 5mM NAC, 5.4 uM Lapatinib, and 1uM ICI alone or in combination in serum-replete media for four days. NAC was administered again on day 3. Cells were collected and apoptosis determined by Annexin V/Propidium Iodide staining. Graphs are representative of 3 biological replicates. BT474 control (**, p = .0017 vs NAC), Lap (**, p = .0013 vs Lap+NAC), Lap+ICI (**, p = .0044 vs triple), MDA-MB-361 ICI (**, p = .0014 vs ICI+NAC), Lap (**, p = .0028 vs Lap+NAC), Lap+ICI (*, p = .0311 vs triple) MCF7 control (**, p = .0055 vs NAC), ICI (**, p = .0096 vs ICI/NAC), Lap/ICI (***, p = .0001 vs triple). Data shown as mean +/- SD of triplicate experiments;* p<0.05, ** p<0.01, ***p<.0.001.

Fig 2 demonstrated that matriptase activation occurs in ER-positive cells in ICI and Lapatinib treated conditions, which we have now identified to be ROS mediated in Fig 5. The first attempts to suppress ICI and Lapatinib-induced matriptase activation with NAC in BT474 cells resulted in cell detachment. We looked to confirm this as cell death in double positive BT474 and MDA-MB-361 cells by comparing levels of cell death via Annexin V/Propidium Iodide staining and flow cytometric analysis following 48 hour ICI, Lapatinib, and NAC treatments alone and in relevant combinations (Fig 6A, 6B). In both cell lines, NAC in combination was more effective at inducing apoptosis than any option than excluded NAC. In BT474 cells, NAC in combination with Lapatinib was more effective than Lapatinib and ICI and the addition of NAC increased the cytotoxicity of Lapatinib and ICI (Fig 6A). In MDA-MB-361 cells, the addition of NAC increased cytotoxicity of Lapatinib and ICI, and while that was less effective than Lapatinib and ICI in combination, the addition of NAC to that combination was the most effective combination (Fig 6B). Previous data has demonstrated matriptase activation in single ER positive MCF7 cells and as such, we also attempted to determine if matriptase-directed NAC treatment alone or in combination could have similar effects on apoptosis (Fig 6C). NAC treatment in MCF7 cells induced apoptosis alone, to a similar degree to ICI. Additionally, the combination of NAC to ICI and ICI and Lapatinib increased the level of apoptosis induced by MCF7 cells (Fig 6C).

### Investigating matriptase-directed 5mM NAC treatment demonstrated increased apoptosis alone or in combination in HER2-amplified breast cancer cells

**Fig 7:**
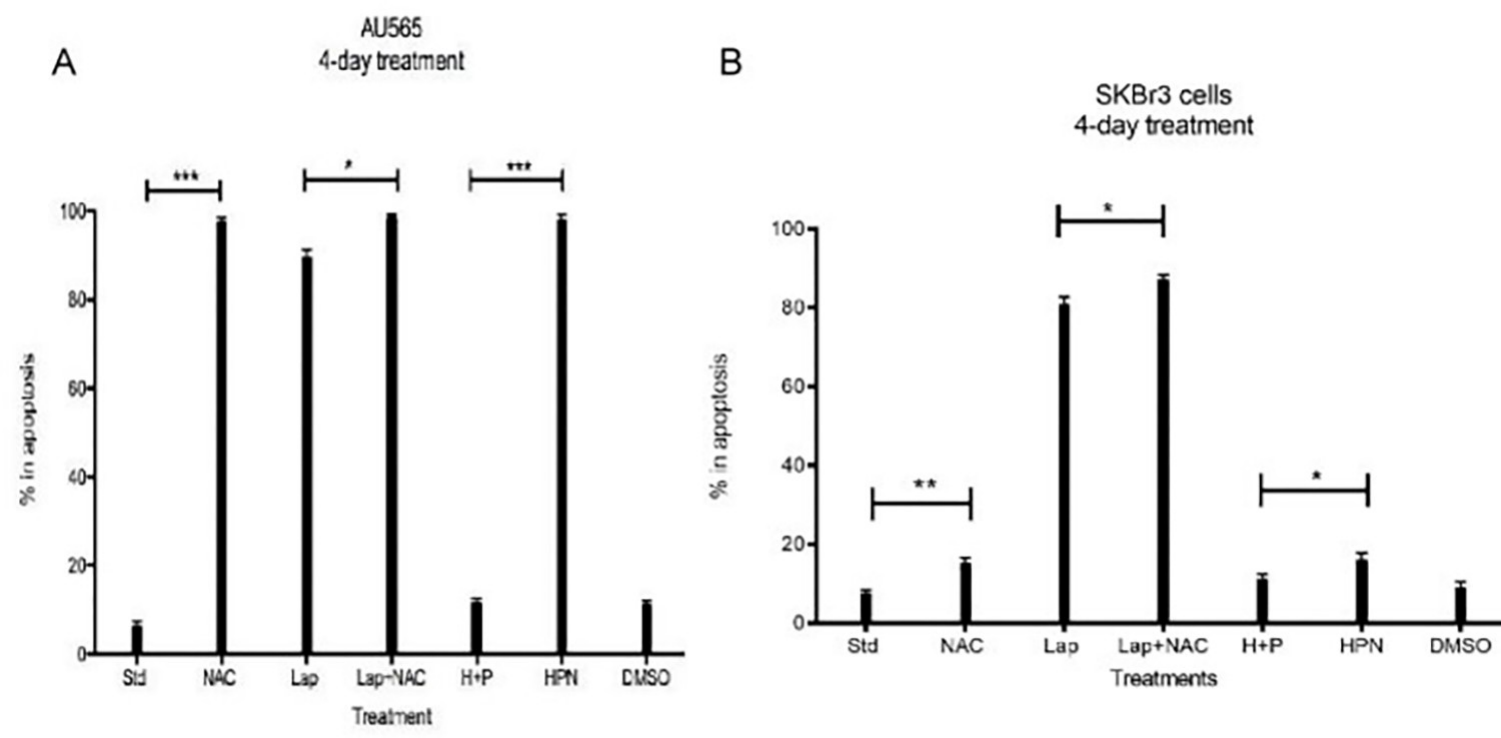
Investigating matriptase-directed 5mM NAC treatment demonstrated increased apoptosis alone or in combination in HER2-amplified breast cancer cells. 100,000 A) AU565 and B) SKBr3 cells were seeded in 6-well plates and allowed to attach overnight. Cells were then treated with 5mM NAC, 5.4 uM Lapatinib and the combination of 10ug/ml Trastuzumab and 10ug/ml Pertuzumab alone or in combination in serum-replete media for four days. NAC was administered again on day 3. Cells were collected and apoptosis determined by Annexin V/Propidium Iodide staining. Graphs are representative of 3 biological replicates. AU565 control (***, p<.001 vs NAC), Lap (*, p = .0159 vs Lap+NAC), H+P(***, p,0.0001), SKBr3 control (**, p = .0034 vs NAC), H+P(*, p = .0299 vs HPN, Lap (*, p = .0134 vs Lap+NAC) (**, p = .0034 vs NAC) Lap^R^ Control (***, p = .0003 vs NAC), MTOR (*, p = .0125 vs combo). Data shown as mean +/- SD of triplicate experiments;* p<0.05, ** p<0.01, ***p<.0.001.

In Fig 2, we have also demonstrated clear matriptase activation in standard growth conditions in two HER2-amplified breast cancer lines and here, we set out to determine the effect of matriptase-directed NAC treatment on this breast cancer subtype. We seeded 100,000 SKBr3 and AU565 cells and after overnight attachment, treated with 5.4uM Lapatinib, 5mM NAC, and 10ug/ml Trastuzumab/Pertuzumab alone or in combination. Cells were then analyzed by Annexin V/Propidium Iodide staining and flow cytometric analysis. In AU565 cells, which demonstrated the highest level of matriptase activation of any cell line in Fig 1, we observe that that 5mM NAC alone was nearly completely cytotoxic alone with no suppression by the addition of other agents (Fig 7A). In SKBR3 cells, which demonstrated a smaller degree of matriptase activation in Fig 2, we observed marginal apoptosis cells treated with NAC alone, with significantly more apoptosis as a result of clinical therapies in combination with NAC. The more effective combination in inducing apoptosis was NAC in combination with Lapatinib (Fig 7B). Trastuzumab and Pertuzumab alone were not effective in inducing apoptosis alone or in combination with NAC.

## Discussion

In this investigation, we have first demonstrated clear matriptase activation due to antiestrogen and Lapatinib treatment in double positive breast cancer cell lines. This was immensely informative and may carry some clinical significance due to their routine clinical use with patients. ICI is often prescribed as second line therapy after treatment with aromatase inhibitors or Tamoxifen have failed [35] and studies, including a clinical trial have included the addition of Lapatinib with ICI. However, in those double positive cells, matriptase activation was low in standard growth conditions and induced only by the addition of an antiestrogen, most commonly ICI, and Lapatinib. This was a novel finding that first suggested the inhibition of ER and EGFR/HER2 signaling pathways induced matriptase activation. Investigating matriptase’s potential contribution in response to ICI and Lapatinib treatment, on survival, migration, invasion or the activation of other growth factor pathways such as HGF/c-MET that have been demonstrated to be stimulated by activated matriptase should follow with the caveat that it should be expected that these cells are in a stressed, and potentially more fragile state.

Here, we also novelly conclude that ROS is central to endogenous and exogenous *in vitro* matriptase activation. This conclusion follows our previous work which demonstrated that heavy metals CdCl2 and CdCl2 and other oxidizing agents such as NEM clearly induce matriptase activation [6, 11] and is novel in its demonstration that ROS-dependent activation is central to even our well-established method of acid induction. The ability of hydrogen peroxide to induce and NAC to suppress matriptase activation demonstrates the central role of ROS in activating matriptase. We postulate that matriptase activation may be an indicator of the levels of oxidative stress experienced by a cell, with high levels of activation following increase of intracellular ROS. This hypothesis could be biologically important as ROS levels play crucial roles in cell function and homeostasis. While excessive ROS levels are lethal in both normal and cancer cells [36, 37], low levels have a crucial role in signaling and the regulation of protein function in cancer cells, such as in the oxidation of reactive cysteines by hydrogen peroxide [26]. The oxidation of cysteines in the active site of phosphatases such as PTEN renders the enzyme inactive and is the mechanism whereby ROS is also able to enhance the duration and magnitude of kinase activity, such as PI3K. Interestingly, it has been shown that EGFR activity also produces ROS [34, 35], particularly hydrogen peroxide and as such, is able to induce kinase activity while inhibiting its regulation. Future work into the plausibility of EGFR-induced matriptase activation, particularly in EGFR-dependent cancers such lung, should follow. Future work should also investigate the mechanism of how intracellular ROS levels modulate levels of matriptase activation should first attempt to determine if oxidation of one or several cysteines in the intracellular domain results in matriptase activation. We have previously shown that matriptase activation increases in a dose-dependent manner with NEM treatment, which is known to oxidize cysteines [4, 30]. There are 34 cysteines in all sequenced domains of matriptase, which does not include amino acids 1-54 of the cytoplasmic tail, suggesting that cysteine oxidation could support an activating conformational change. It is possible that matriptase’s requirement for autoactivation may be due to the requirement of sufficient oxidation of intracellular residues to initiate a conformational in its serine protease domain. There are six cysteines needed to form three disulfide bridge that stabilize matriptase’s catalystic pocket within the serine protease domain. The most likely cysteine pairings are: Cys-469–Cys-485, Cys-604–Cys-618, and Cys-629–Cys-658 and are excellent candidates for future study [38]. Additionally there is a predicted disulfide linkage between two cysteine residues corresponding to Cys604 and Cys731 in full-length matriptase that could be considered [39].

Another unexpected finding of this investigation was the effectiveness of matriptase activation to foreshadow the efficacy of NAC-induced apoptosis in cell lines. In cell lines across clinical breast cancer subtypes, we observe that using matriptase activation to direct NAC treatment has a nearly complete cytotoxic effect when in combination with kinase inhibition. These findings support the literature that demonstrated intracellular ROS can have a tumor pro-survival effect [40] and we have novelly shown that effect is separate from the effects of kinase pathway inhibition. The efficacy of NAC in combination with clinical inhibitors in inducing apoptosis demonstrates that ROS-mediated survival contributes to overall survival separate from kinase-mediated survival. In tested breast cancers, we observed an additive induction of apoptosis from the combination with NAC. We interpret the difference in apoptosis in response to single NAC and combination treatment as a difference in the cell’s dependence on ROS-mediated as well as kinase-mediated survival. While we know that previous literature has shown that NAC therapy has had varying degrees of antitumor efficacy in patients, the conditions and tight regulation of matriptase activation may be a link to understanding the cellular conditions where NAC therapy could be effective. Further testing should first include the use of ROS reporter system to be able to assess ROS levels in parallel with matriptase activation. These data could be useful in understanding if matriptase activation happens in a window or perhaps, after a threshold level of intracellular ROS is reached as well as if the intracellular ROS must localized to a particular area of the cell. Additional testing should include more examples of each type of tested cancers as well as an expanded investigation into all epithelial cancers, with the caveat that matriptase expression, and therefore this window into ROS-mediated survival, is lacking in more mesenchymal-like cancers. In those cell types, another means of assessing ROS, perhaps the aforementioned ROS reporter system could be useful in indicating potential NAC therapy. Future work should also include understanding the mechanism by which NAC is inducing cell death in these cells. With two known mechanisms of its activity, it is unclear which is directly responsible for the cell death we have observed. Future work should also consider if matriptase activation is suppressed by another antioxidant and its effect on cell death alone or in combination.

Since the early 1990s, our lab has studied the mechanism and effects of matriptase activation in normal and cancer cell contexts. We have learned much from our and collaborative study of the protease, such as its robust induction by decrease in intracellular pH [11], inflammatory conditions [6] , and its ability to act as an oncogene and tumor promotor [17, 41], however, the central mechanism has remained unclear. In this investigation, we have novelty implicated intracellular ROS, particularly hydrogen peroxide, as the central player in mechanism of endogenous and acid-induced matriptase activation.

## Supporting information

S1 File (PDF)

## References

[1] Tseng I.-C. et al., “Purification from human milk of matriptase complexes with secreted serpins: mechanism for inhibition of matriptase other than HAI-1.,” *Am. J. Physiol. Cell Physiol.*, vol. 295, no. 2, pp. C423–31, Aug. 2008. 18550704 10.1152/ajpcell.00164.2008 PMC2518410

[2] Oberst M. D. et al., “HAI-1 regulates activation and expression of matriptase, a membrane-bound serine protease,” *Am. J. Physiol*. Physiol., vol. 289, no. 2, pp. C462–C470, Aug. 2005. 10.1152/ajpcell.00076.2005 15800053

[3] Chen C.-J. et al., “Increased matriptase zymogen activation in inflammatory skin disorders.,” *Am. J. Physiol. Cell Physiol.*, vol. 300, no. 3, pp. C406–15, Mar. 2011. 21123732 10.1152/ajpcell.00403.2010 PMC3063967

[4] Lee M.-S. et al., “Autoactivation of matriptase in vitro: requirement for biomembrane and LDL receptor domain.,” *Am. J. Physiol. Cell Physiol.*, vol. 293, no. 1, pp. C95–105, Jul. 2007. 17344310 10.1152/ajpcell.00611.2006

[5] Sun P. et al., “Decreasing the ratio of matriptase/HAI‑1 by downregulation of matriptase as a potential adjuvant therapy in ovarian cancer.,” *Mol. Med. Rep.*, vol. 14, no. 2, pp. 1465–74, Aug. 2016. 27356668 10.3892/mmr.2016.5435 PMC4940087

[6] Wang J.-K. et al., “Matriptase autoactivation is tightly regulated by the cellular chemical environments.,” *PLoS One*, vol. 9, no. 4, p. e93899, Jan. 2014. 24705933 10.1371/journal.pone.0093899 PMC3976350

[7] Chu L.-L. et al., “Human cancer cells retain modest levels of enzymatically active matriptase only in extracellular milieu following induction of zymogen activation.,” *PLoS One*, vol. 9, no. 3, p. e92244, Jan. 2014. 24663123 10.1371/journal.pone.0092244 PMC3963879

[8] Lee M.-S., Kiyomiya K., Benaud C., Dickson R. B., and Lin C.-Y., “Simultaneous activation and hepatocyte growth factor activator inhibitor 1-mediated inhibition of matriptase induced at activation foci in human mammary epithelial cells,” *Am. J. Physiol. Physiol*., vol. 288, no. 4, pp. C932–C941, Apr. 2005. 10.1152/ajpcell.00497.2004 15590895

[9] Wu S.-R. et al., “Matriptase is involved in ErbB-2-induced prostate cancer cell invasion.,” *Am. J. Pathol.*, vol. 177, no. 6, pp. 3145–58, Dec. 2010. 20971737 10.2353/ajpath.2010.100228 PMC2993294

[10] Ko C.-J. et al., “Androgen-Induced TMPRSS2 Activates Matriptase and Promotes Extracellular Matrix Degradation, Prostate Cancer Cell Invasion, Tumor Growth, and Metastasis,” *Cancer Res.*, vol. 75, no. 14, 2015. 10.1158/0008-5472.CAN-14-3297 26018085

[11] Tseng I.-C. et al., “Matriptase activation, an early cellular response to acidosis.,” *J. Biol. Chem.*, vol. 285, no. 5, pp. 3261–70, Jan. 2010. 19940125 10.1074/jbc.M109.055640 PMC2823413

[12] Tseng C. C. et al., “Matriptase shedding is closely coupled with matriptase zymogen activation and requires de novo proteolytic cleavage likely involving its own activity,” *PLoS One*, vol. 12, no. 8, Aug. 2017. 10.1371/journal.pone.0183507 PMC5567652 28829816

[13] List K. et al., “Epithelial integrity is maintained by a matriptase-dependent proteolytic pathway.,” *Am. J. Pathol.*, vol. 175, no. 4, pp. 1453–63, Oct. 2009. 19717635 10.2353/ajpath.2009.090240 PMC2751542

[14] Lee S.-L., Huang P.-Y., Roller P., Cho E.-G., Park D., and Dickson R. B., “Matriptase/epithin participates in mammary epithelial cell growth and morphogenesis through HGF activation.,” *Mech. Dev.*, vol. 127, no. 1–2, pp. 82–95, Jan. 2010. 19853659 10.1016/j.mod.2009.10.004

[15] Basel-Vanagaite L. et al., “Autosomal recessive ichthyosis with hypotrichosis caused by a mutation in ST14, encoding type II transmembrane serine protease matriptase,” *Am. J. Hum. Genet.*, vol. 80, no. 3, pp. 467–477, 2007. 17273967 10.1086/512487 PMC1821100

[16] Chen Y.-W. et al., “Matriptase regulates proliferation and early, but not terminal, differentiation of human keratinocytes.,” *J. Invest. Dermatol.*, vol. 134, no. 2, pp. 405–414, Feb. 2014. 23900022 10.1038/jid.2013.320 PMC3925676

[17] List K. et al., “Deregulated matriptase causes ras-independent multistage carcinogenesis and promotes ras-mediated malignant transformation.,” *Genes Dev.*, vol. 19, no. 16, pp. 1934–50, Aug. 2005. 16103220 10.1101/gad.1300705 PMC1186192

[18] Zoratti G. L. et al., “Matriptase regulates c-Met mediated proliferation and invasion in inflammatory breast cancer,” *Oncotarget*, vol. 7, no. 36, pp. 58162–58173, Sep. 2016. 27528224 10.18632/oncotarget.11262 PMC5295421

[19] Le Gall S. M. et al., “Matriptase activation connects tissue factor-dependent coagulation initiation to epithelial proteolysis and signaling.,” *Blood*, vol. 127, no. 25, pp. 3260–9, 2016. 27114461 10.1182/blood-2015-11-683110 PMC4920024

[20] Bardou O. et al., “Membrane-anchored Serine Protease Matriptase Is a Trigger of Pulmonary Fibrogenesis,” *Am. J. Respir. Crit. Care Med.*, vol. 193, no. 8, pp. 847–860, Apr. 2016. 26599507 10.1164/rccm.201502-0299OC PMC4849177

[21] Waks A. G. and Winer E. P., “Breast Cancer Treatment,” *JAMA*, vol. 321, no. 3, p. 288, Jan. 2019. 30667505 10.1001/jama.2018.19323

[22] Hubalek M., Czech T., and Müller H., “Biological Subtypes of Triple-Negative Breast Cancer.,” *Breast Care (Basel)*., vol. 12, no. 1, pp. 8–14, Mar. 2017. 28611535 10.1159/000455820 PMC5465739

[23] Lu W., Ogasawara M. A., and Huang P., “Models of reactive oxygen species in cancer.,” *Drug Discov. Today. Dis. Models*, vol. 4, no. 2, pp. 67–73, 2007. 18591999 10.1016/j.ddmod.2007.10.005 PMC2390932

[24] Wallace D. C., “Mitochondria and cancer.,” *Nat. Rev. Cancer*, vol. 12, no. 10, pp. 685–98, Oct. 2012. 23001348 10.1038/nrc3365 PMC4371788

[25] Zhou D., Shao L., and Spitz D. R., “Reactive oxygen species in normal and tumor stem cells.,” *Adv. Cancer Res.*, vol. 122, pp. 1–67, 2014. 24974178 10.1016/B978-0-12-420117-0.00001-3 PMC4207279

[26] Ray P. D., Huang B.-W., and Tsuji Y., “Reactive oxygen species (ROS) homeostasis and redox regulation in cellular signaling.,” *Cell. Signal.*, vol. 24, no. 5, pp. 981–90, May 2012. 22286106 10.1016/j.cellsig.2012.01.008 PMC3454471

[27] Zhang S. et al., “Dynamics of estrogen-induced ROS and DNA strand break generation in estrogen receptor α-positive breast cancer,” 2022. 10.1016/j.bbrc.2022.02.089 35278890

[28] Perillo B., Di Santi A., Cernera G., O. M. N, G. Castoria, and A. Migliaccio, “Phosphorylation of H3 serine 10 by IKKα governs cyclical production of ROS in estrogen-induced transcription and ensures DNA wholeness.” *Cell Death Differ*. 2014 Sep; 21(9): 1503. 24971480 10.1038/cdd.2014.91 PMC4131185

[29] Lee M.-S., Kiyomiya K., Benaud C., Dickson R. B., and Lin C.-Y., “Simultaneous activation and hepatocyte growth factor activator inhibitor 1-mediated inhibition of matriptase induced at activation foci in human mammary epithelial cells.,” *Am. J. Physiol. Cell Physiol.*, vol. 288, no. 4, pp. C932–41, Apr. 2005. 15590895 10.1152/ajpcell.00497.2004

[30] Oberst M. D., Williams C. A., Dickson R. B., Johnson M. D., and Lin C.-Y., “The activation of matriptase requires its noncatalytic domains, serine protease domain, and its cognate inhibitor.,” *J. Biol. Chem.*, vol. 278, no. 29, pp. 26773–9, Jul. 2003. 12738778 10.1074/jbc.M304282200

[31] D’Amato V. et al., “Mechanisms of lapatinib resistance in HER2-driven breast cancer,” *Cancer Treat. Rev.*, vol. 41, no. 10, pp. 877–883, Dec. 2015. 26276735 10.1016/j.ctrv.2015.08.001

[32] Ngan R. K. C., “Management of hormone-receptor positive human epidermal receptor 2 negative advanced or metastatic breast cancers,” *Ann. Transl. Med.*, vol. 6, no. 14, pp. 284–284, Jul. 2018. doi: 10.1007/s10549-021-06383-5 30105234 PMC6068330

[33] Shin H.-J., Kwon H.-K., Lee J.-H., Anwar M. A., and Choi S., “Etoposide induced cytotoxicity mediated by ROS and ERK in human kidney proximal tubule cells,” *Sci. Rep.*, vol. 6, no. 1, p. 34064, Dec. 2016. doi: 10.1038/srep34064 27666530 PMC5036097

[34] Ueno N. T. and Mamounas E. P., “Neoadjuvant nab-paclitaxel in the treatment of breast cancer.,” *Breast Cancer Res. Treat.*, vol. 156, no. 3, pp. 427–440, Apr. 2016. 27072366 10.1007/s10549-016-3778-z PMC4837202

[35] Mills J. N., Rutkovsky A. C., and Giordano A., “Mechanisms of resistance in estrogen receptor positive breast cancer: overcoming resistance to tamoxifen/aromatase inhibitors,” *Curr. Opin. Pharmacol.*, vol. 41, pp. 59–65, Aug. 2018. 29719270 10.1016/j.coph.2018.04.009 PMC6454890

[36] Weng M.-S., Chang J.-H., Hung W.-Y., Yang Y.-C., and Chien M.-H., “The interplay of reactive oxygen species and the epidermal growth factor receptor in tumor progression and drug resistance.,” *J. Exp. Clin. Cancer Res.*, vol. 37, no. 1, p. 61, Mar. 2018. 29548337 10.1186/s13046-018-0728-0 PMC5857086

[37] Heppner D. E. and van der Vliet A., “Redox-dependent regulation of epidermal growth factor receptor signaling,” *Redox Biol.*, vol. 8, pp. 24–27, 2016. 26722841 10.1016/j.redox.2015.12.002 PMC4710793

[38] C.-Y. Lin, J. Anders, M. D. Johnson, Q. A. Sang, and R. B. Dickson, “Molecular Cloning of cDNA for Matriptase, a Matrix-degrading Serine Protease with Trypsin-like Activity* Title.” 10.1074/jbc.274.26.18231 10373424

[39] Inouye K. et al., “Roles of CUB and LDL receptor class A domain repeats of a transmembrane serine protease matriptase in its zymogen activation.,” *J. Biochem.*, vol. 153, no. 1, pp. 51–61, Jan. 2013. 23038671 10.1093/jb/mvs118 PMC3527997

[40] Schumacker P. T., “Reactive oxygen species in cancer cells: Live by the sword, die by the sword,” *Cancer Cell*, vol. 10, no. 3, pp. 175–176, Sep. 2006. 16959608 10.1016/j.ccr.2006.08.015

[41] Sales K. U. et al., “Non-hematopoietic PAR-2 is essential for matriptase-driven pre-malignant progression and potentiation of ras-mediated squamous cell carcinogenesis,” *Oncogene*, vol. 34, no. 3, pp. 346–356, Jan. 2014. 24469043 10.1038/onc.2013.563 PMC4112178

