# Supplementary material for: ROS is a master regulator of in vitro matriptase activation
Source: PLoS One. 2023 Jan 30;18(1):e0267492. doi: 10.1371/journal.pone.0267492 (PMC9886240; doi:10.1371/journal.pone.0267492)

Supporting information

Figure 2A Raw images. Breast cancer panel conditioned media screen – 15ul per lane

KD

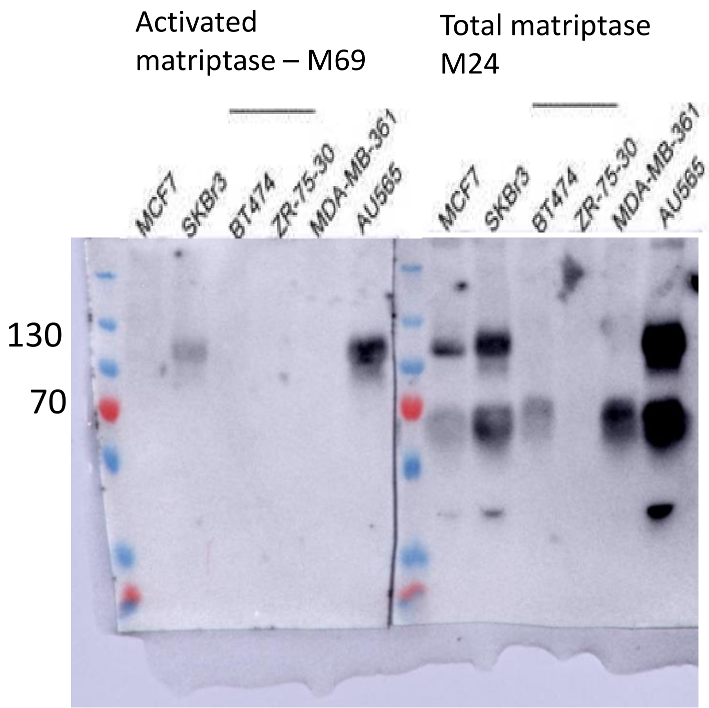

Figure 2A Raw images: BT474 cell conditioned media 15ul per lane

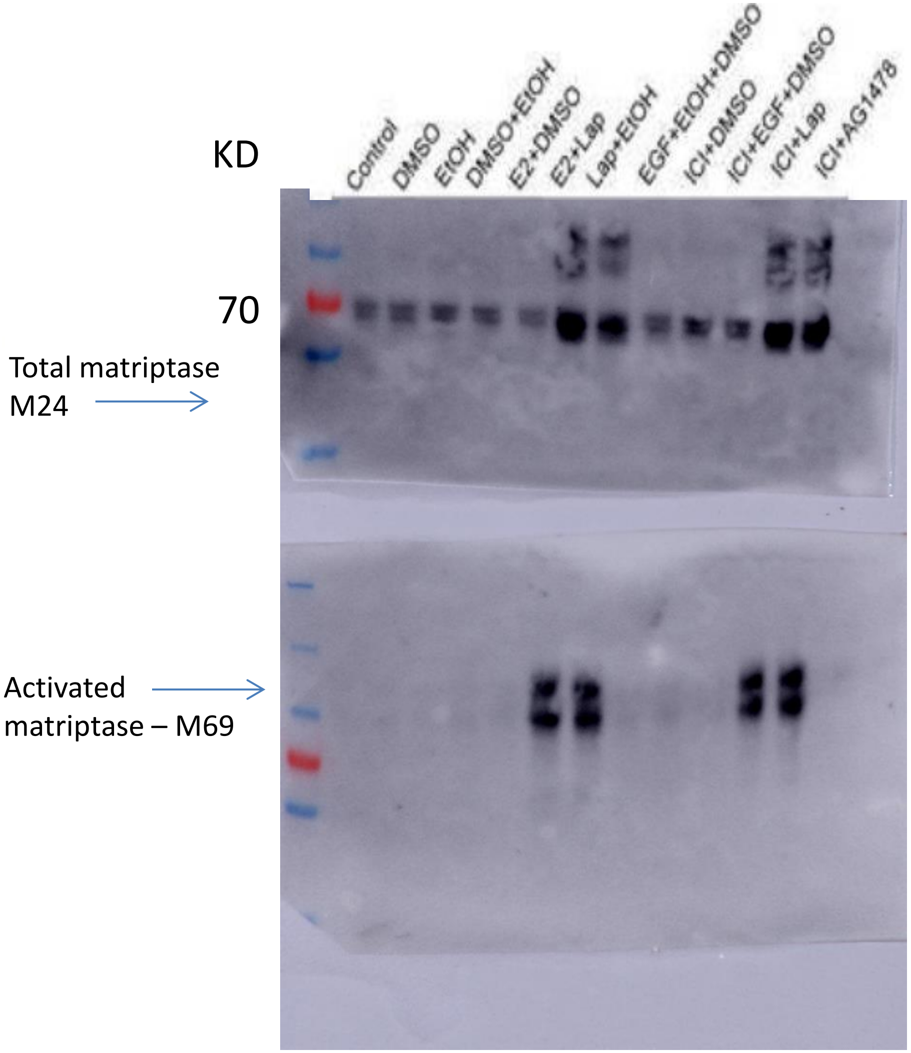

Figure 3A Raw images. BT474 screen – 15ul per lane

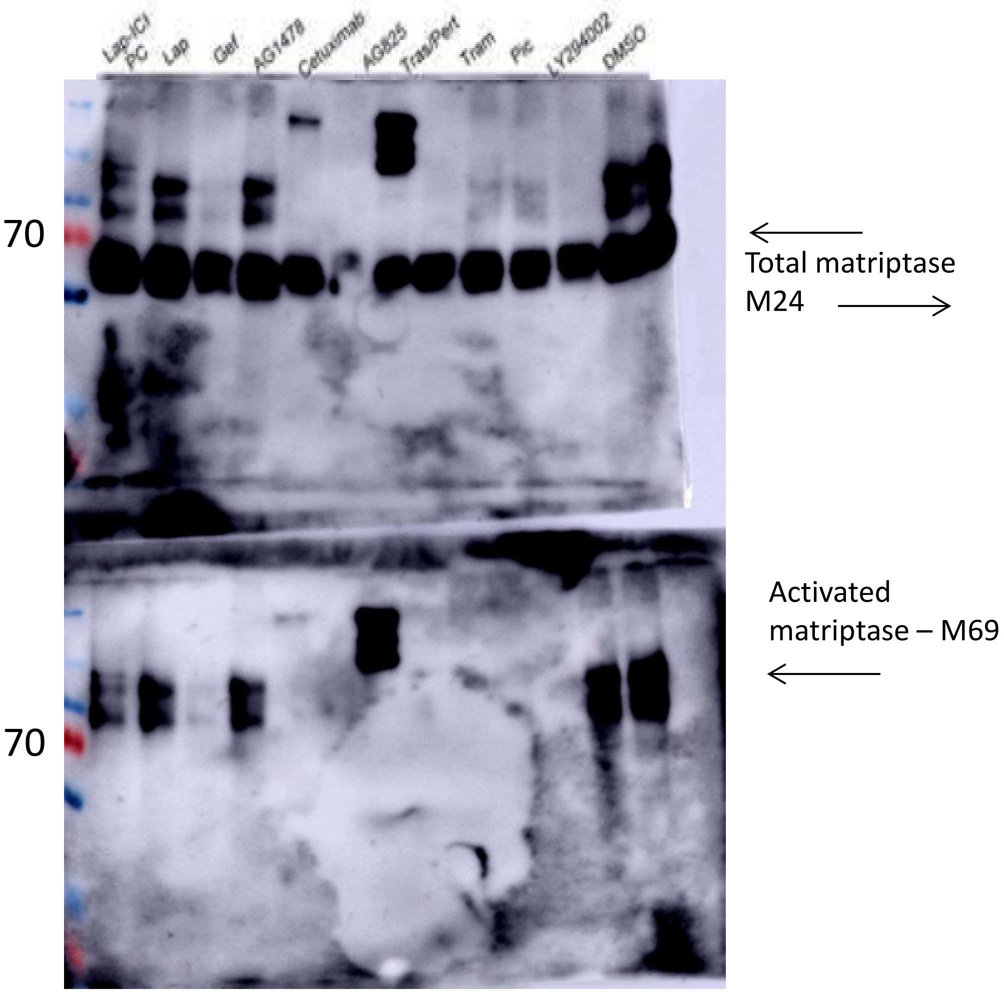

Figure 3B Raw images. ZR-75-30 consolidated screen– 15ul per lane

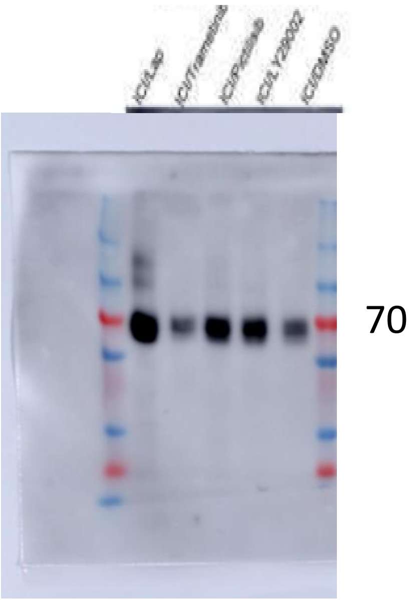

Figure 3B. Raw images. BT474 screen – 15ul per lane

70

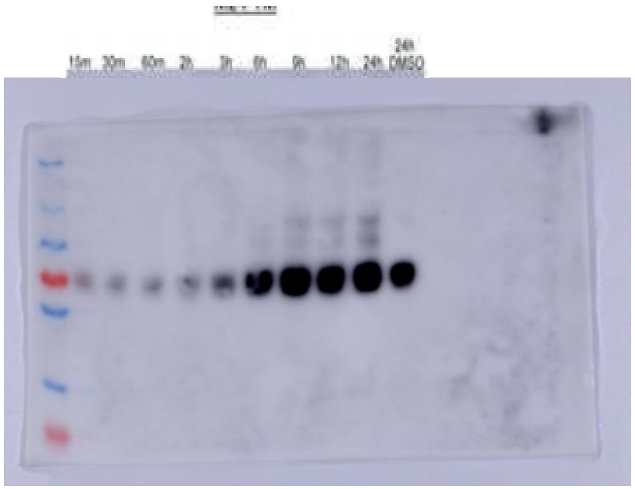

Total matriptase – M24

70

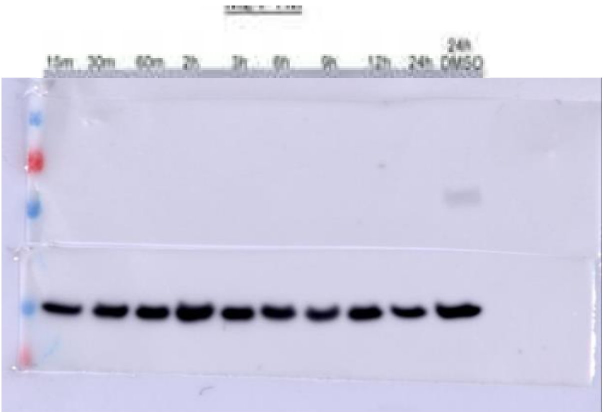

P-AKT

GAPDH

70

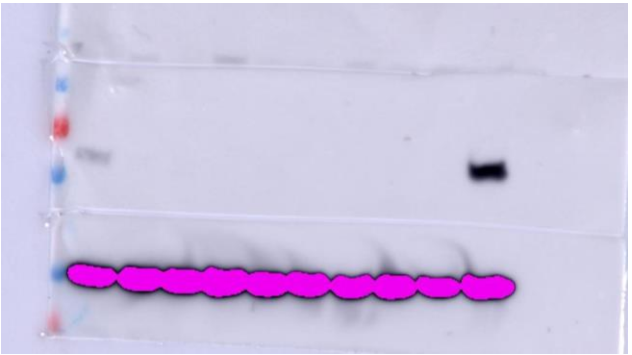

P-AKT

GAPDH

Figure 4B. Raw data/images. BT474 screen – 15ul per lane

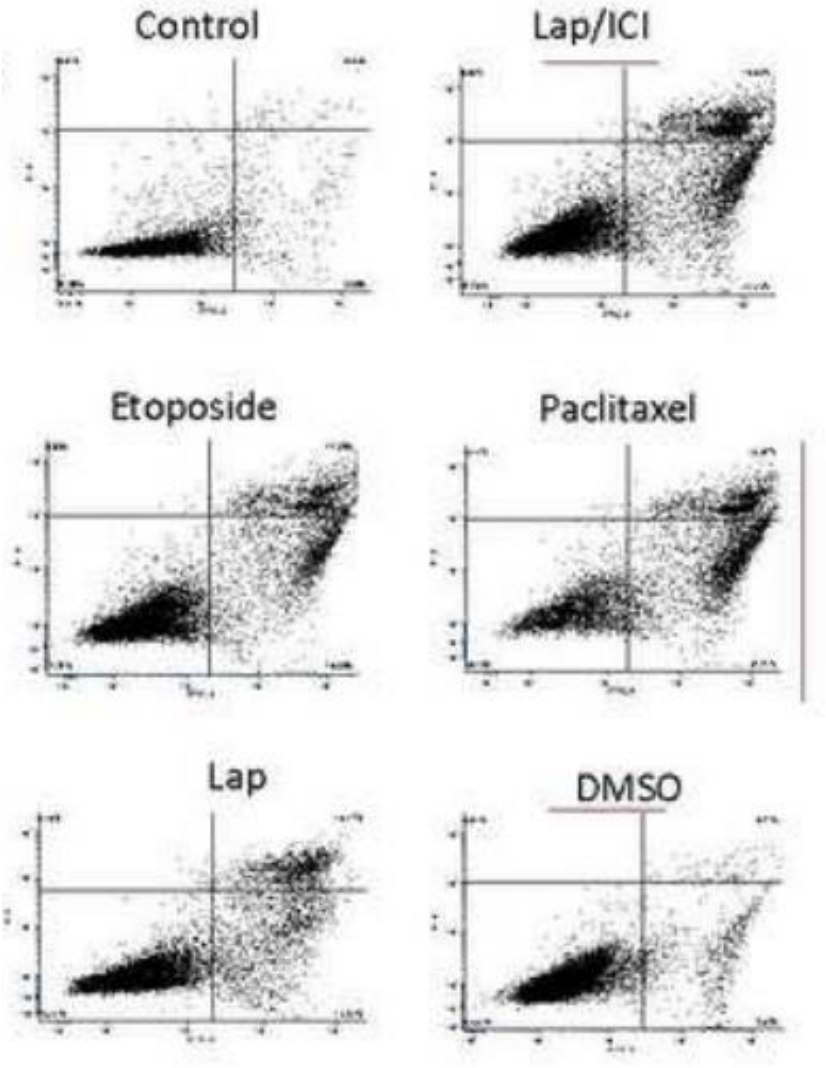

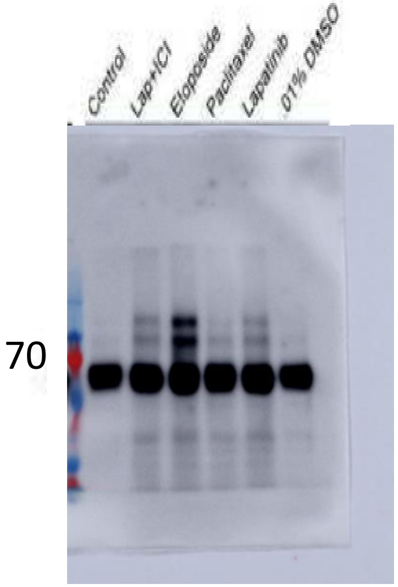

Total matriptase – M24

Figure 5. Raw data/images.

A

Total matriptase – M24

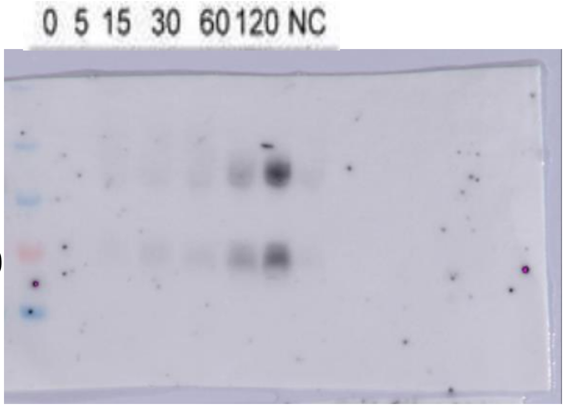

70

SkBr3
Hydrogen peroxide induction

B

Total matriptase – M24

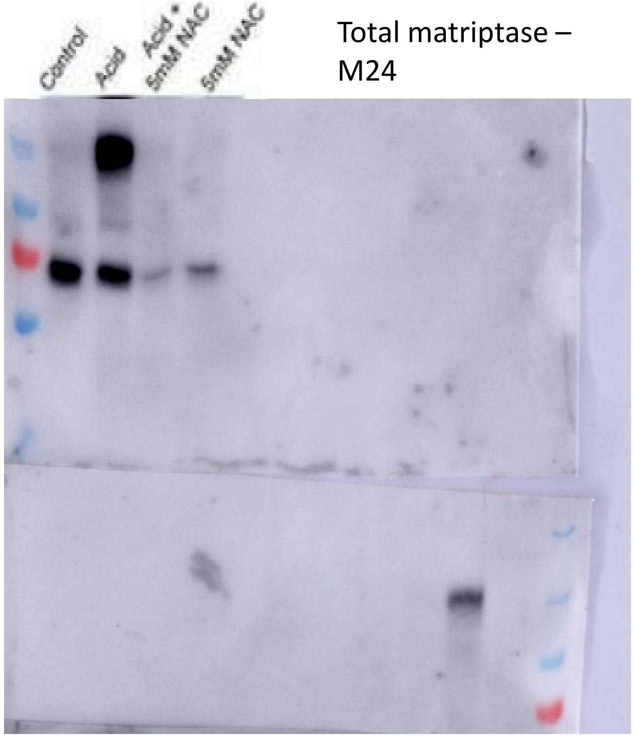

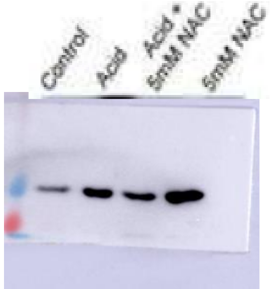

GAPDH

Acid induction and NAC treatment – BT474 cells

Activated matriptase – M69

C

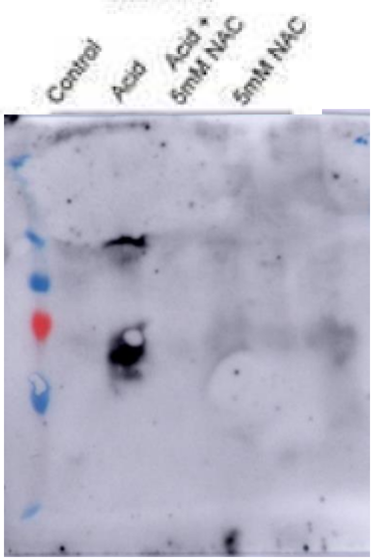

BT474 acid induction test
Hydrogen peroxideand NAC

Figure 5. Raw data/images.

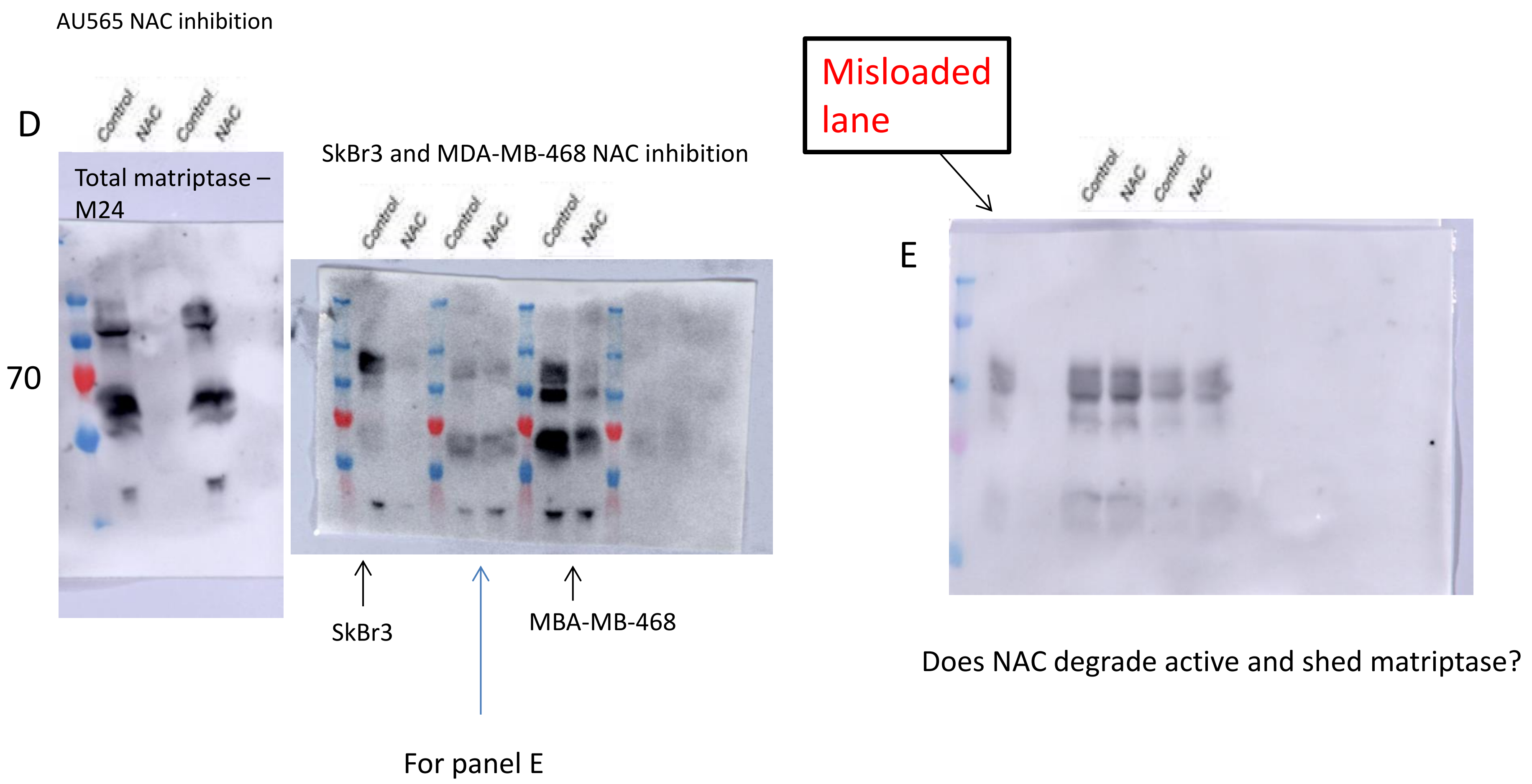

Figure 6. Raw data/images.

Annexin V data and respective graph

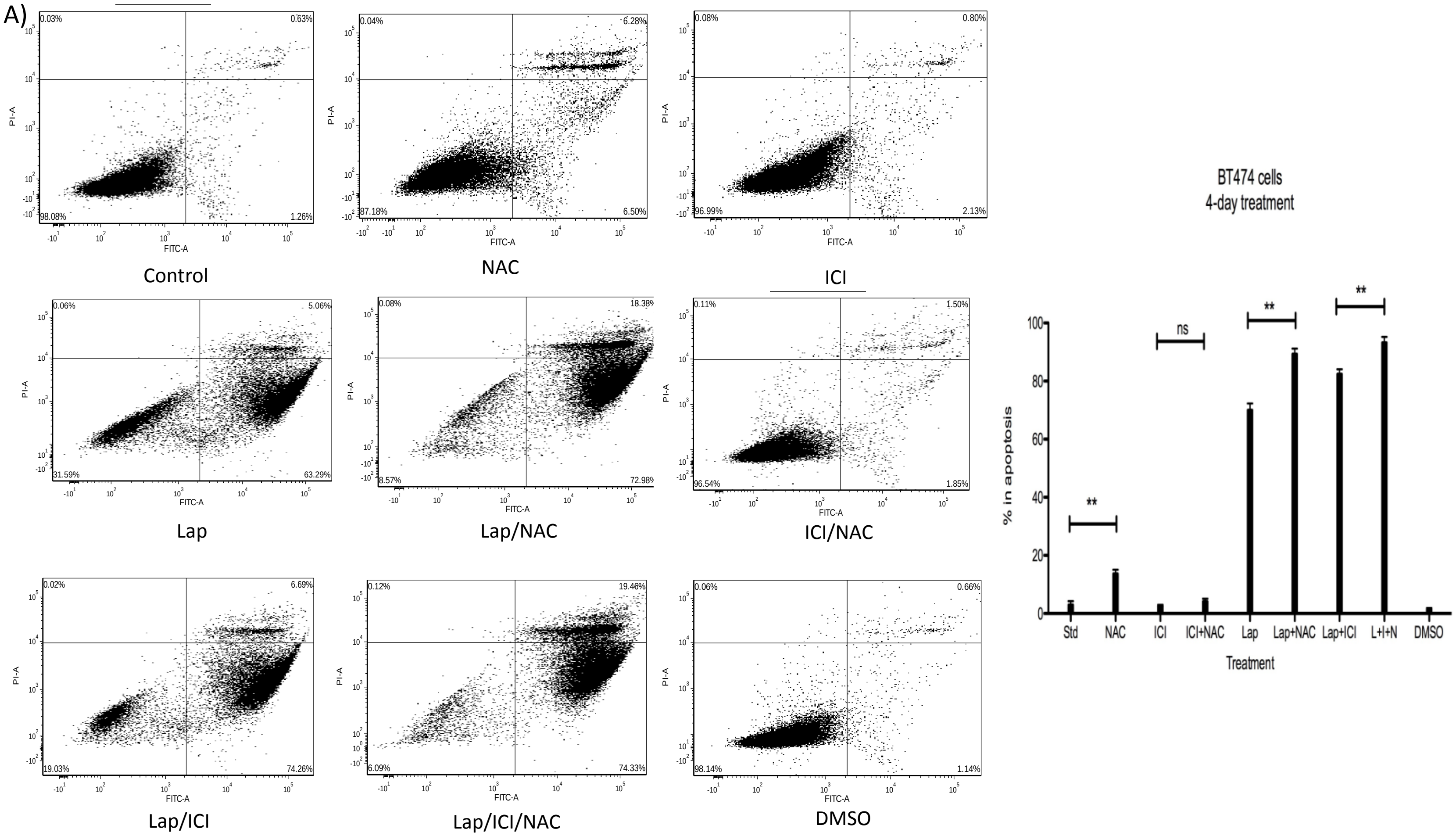

Figure 6. Raw data/images.

Annexin V data and respective graph

B)

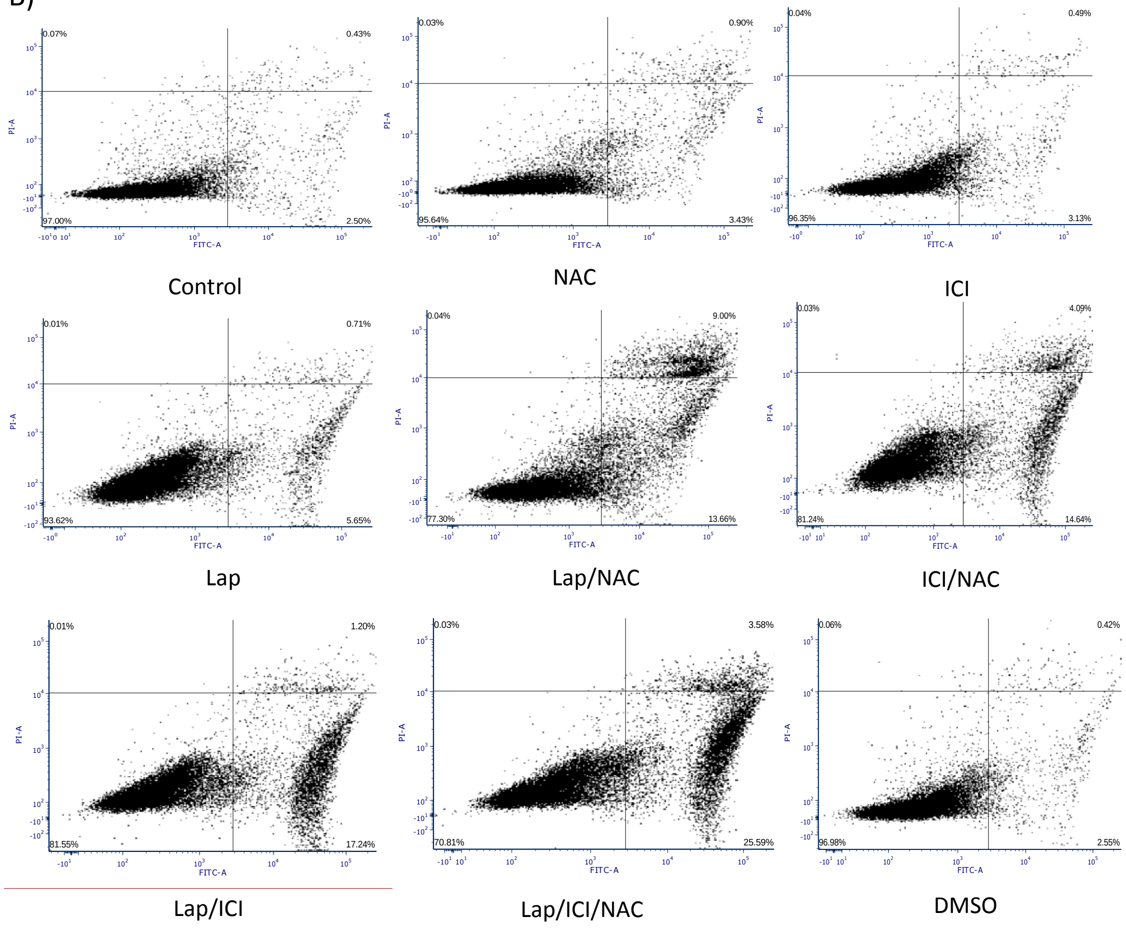

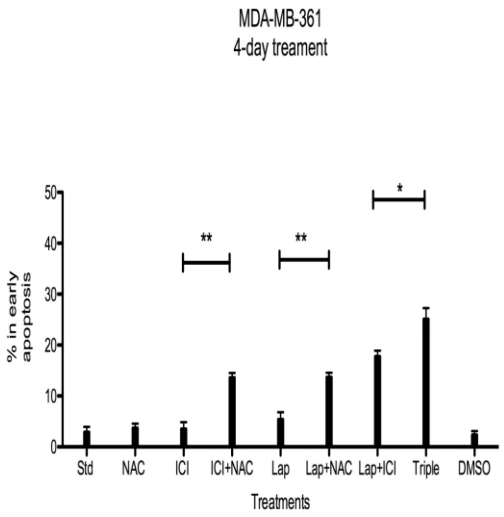

Figure 6. Raw data/images.

Annexin V data and respective graph

C)

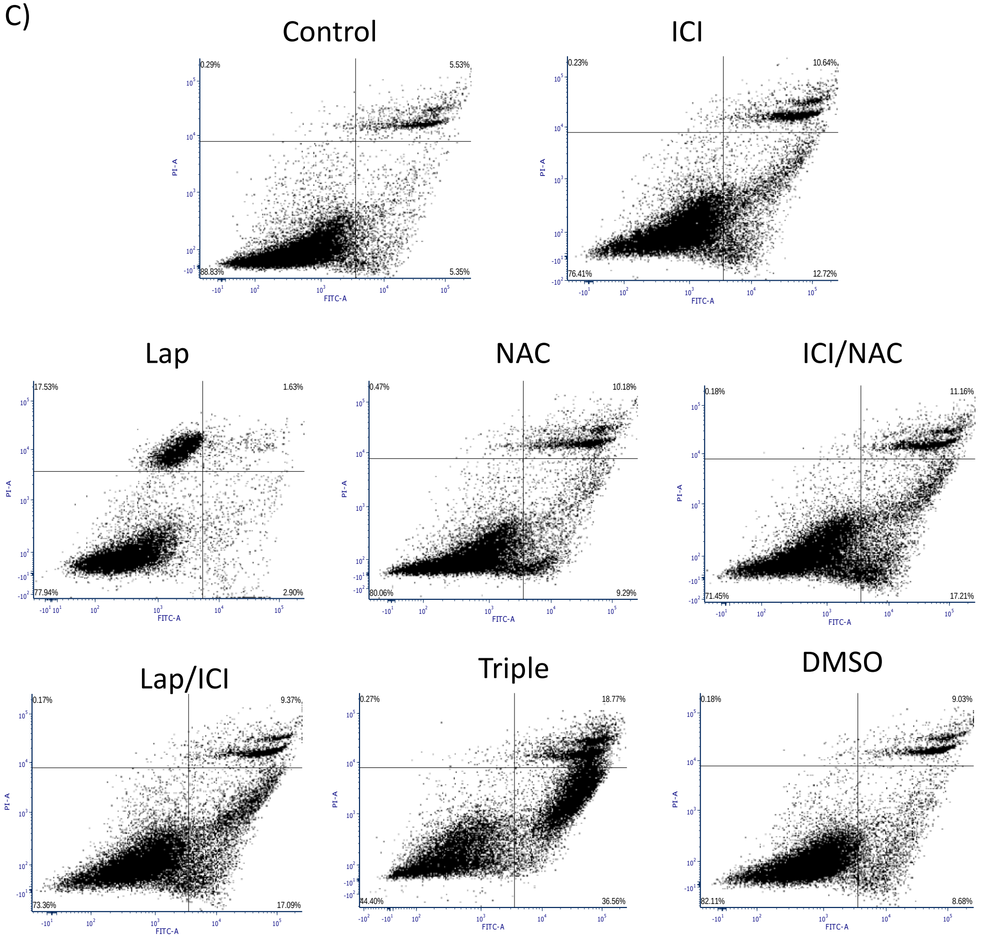

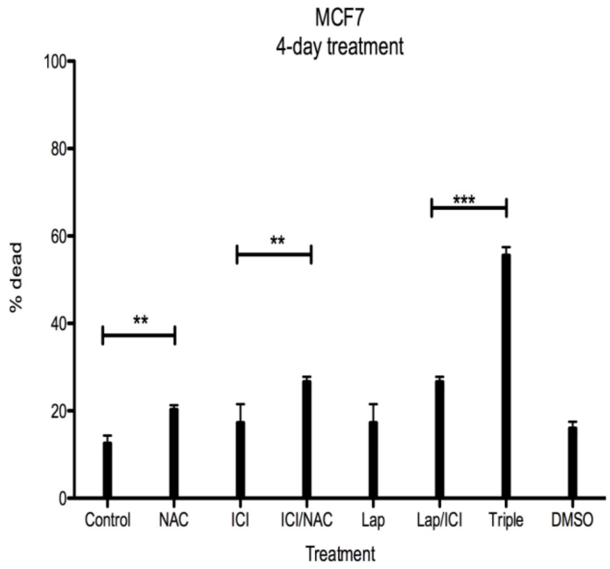

Figure 7. Raw data/images.

Annexin V data and respective graph

A)

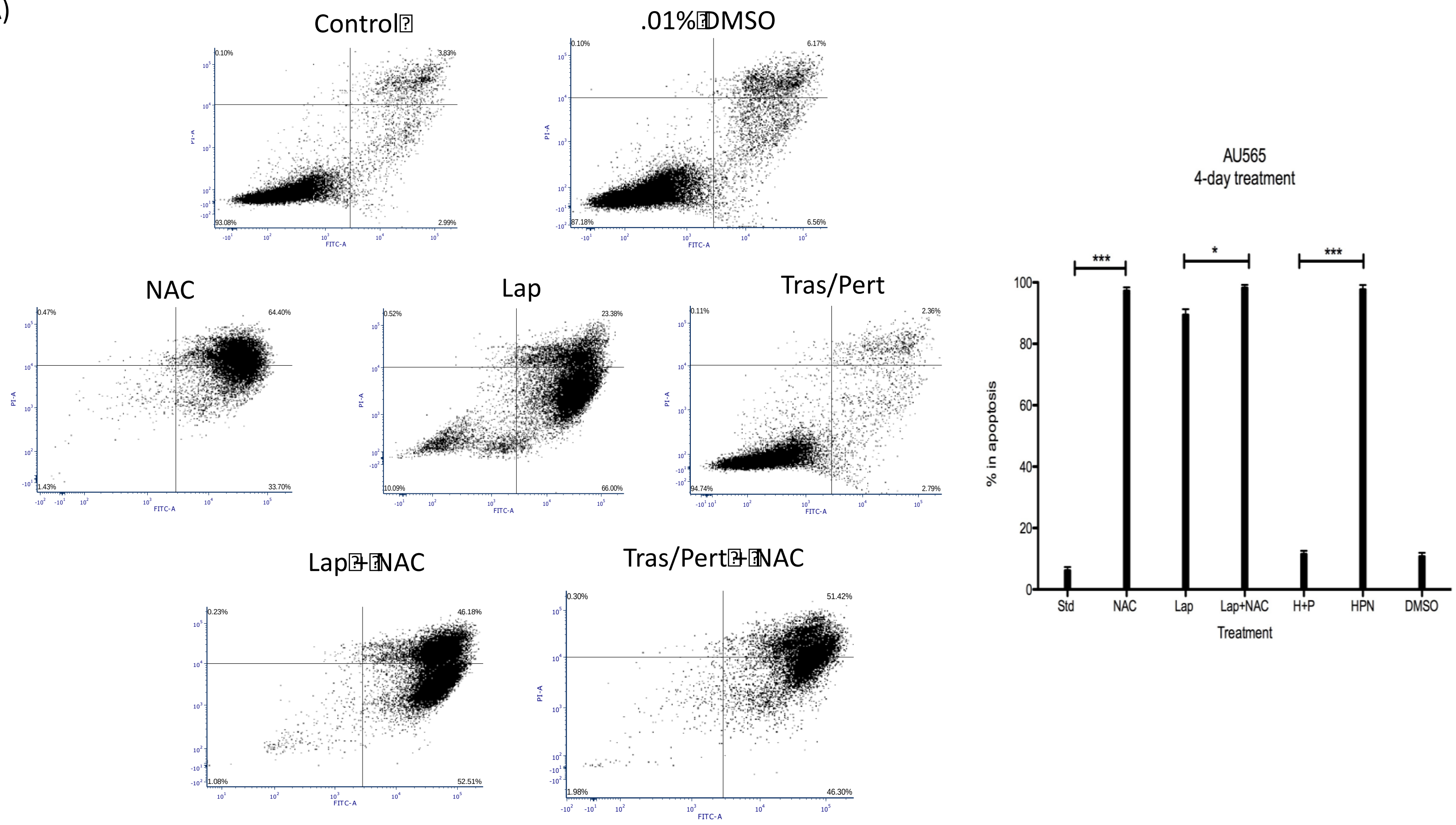

Figure 7. Raw data/images.

Annexin V data and respective graph

B)

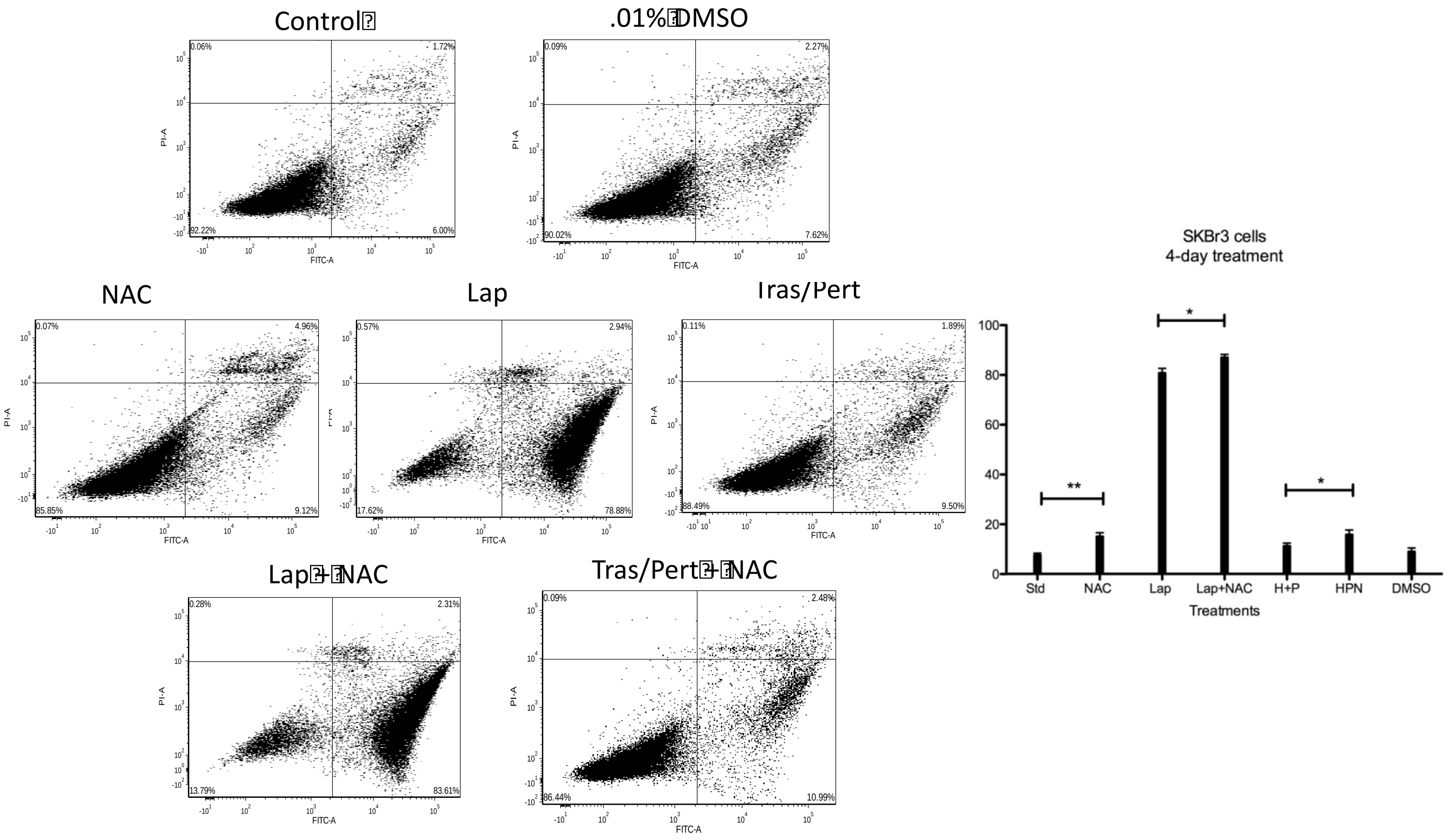

Supplement: S1 File — (PDF) [file pone.0267492.s001.pdf]
